# Enhanced Maize Leaf Disease Detection and Classification Using an Integrated CNN‐ViT Model

**DOI:** 10.1002/fsn3.70513

**Published:** 2025-06-30

**Authors:** Gunjan Shandilya, Sheifali Gupta, Heba G. Mohamed, Salil Bharany, Ateeq Ur Rehman, Seada Hussen

**Affiliations:** ^1^ Chitkara University Institute of Engineering and Technology Chitkara University Punjab India; ^2^ Department of Electrical Engineering, College of Engineering Princess Nourah Bint Abdulrahman University Riyadh Saudi Arabia; ^3^ School of Computing Gachon University Seongnam‐Si Republic of Korea; ^4^ Department of Electrical Power Adama Science and Technology University Adama Ethiopia

**Keywords:** CD&S dataset, convolutional neural network (CNN), deep learning (DL), hybrid CNN‐ViT, maize leaf disease, plant disease classification, plant village dataset, vision transformer (ViT)

## Abstract

Maize crop productivity is significantly impacted by various foliar diseases, emphasizing the need for early, accurate, and automated disease detection methods to enable timely intervention and ensure optimal crop management. Traditional classification techniques often fall short in capturing the complex visual patterns inherent in disease‐affected leaf imagery, resulting in limited diagnostic performance. To overcome these limitations, this study introduces a robust hybrid deep learning framework that synergistically combines convolutional neural networks (CNNs) and vision transformers (ViTs) for enhanced maize leaf disease classification. In the proposed architecture, the CNN module effectively extracts fine‐grained local features, while the ViT module captures long‐range contextual dependencies through self‐attention mechanisms. The complementary features obtained from both branches are concatenated and passed through fully connected layers for final classification. Data from Mendeley and Kaggle were used to build and check the model, and the model did this by applying image resizing, data normalization, expanding its training data, and shuffling the data to increase generalization. Additional testing is done on the corn disease and severity (CD&S) dataset, which is separate from the main combined dataset. After validation, the accuracy of the proposed model was 99.15%, and each of its precision, recall, and F1‐score equaled 99.13%. To confirm it is statistically reliable, 5‐fold cross‐validation was performed, reporting on the Kaggle + Mendeley set an average accuracy of 99.06% and on the CD&S dataset 95.93%. As both of these scores are high, it shows that the model works well across other datasets as well. Experiments have shown that Hybrid CNN‐ViT works better than standalone CNNs. Dropout regularization and using the RAdam optimizer greatly improved both stability and performance. The model stood out as a reliable, high‐accuracy method for discovering maize diseases correctly, which may be valuable in real agricultural settings.

## Graphical abstract

A hybrid CNN‐ViT model was proposed to improve maize leaf disease classification by leveraging both local and global image features. The model achieved 99.15% accuracy, with precision, recall, and F1‐score all at 99.13%, outperforming standalone CNN and ViT architectures. Cross‐validation and testing on external datasets confirmed the model's robustness and potential for real‐world agricultural deployment.

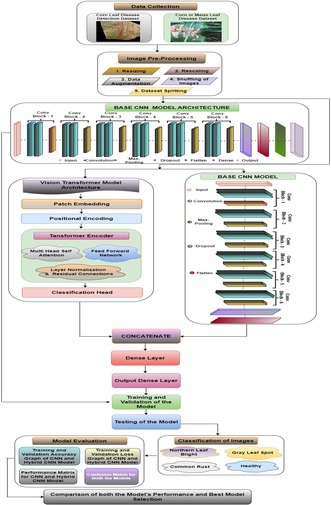

## 1 Introduction

The crop, known as maize or corn, is extremely important worldwide because of its contributions to food, industry, and economic progress. Stopping in the Balsas area of southwest Mexico some 9000 years ago, maize has become an important crop produced worldwide. The United States is number one in production, while India comes in seventh in the world and fourth in the size of its cultivated crops (Rangarajan Aravind et al. 2020; Dash et al. 2023). In India, almost all maize (83%) is grown in the Kharif season, whereas only a minor amount (17%) is produced in the Rabi season. This crop serves many uses, including providing animal feed, making starch, producing processed food, and being traded internationally, and together, they make up about 47% of its domestic use (Sahu and Amudha 2022). Major problems for maize production include many foliar diseases that can considerably lower the quantity and quality of the crop. In India, the common maize diseases, common rust (CR), gray leaf spot (GLS), and northern leaf blight (NLB), account for approximately 14.6% of lower crop yields. Because of these problems, there is a pressing need for methods to quickly detect diseases so crop cultivation can be sustained. Currently, farmers and agricultural experts depend on manual observations to diagnose diseases, but this method is slow, based on opinions, and can include mistakes. In addition, current methods cannot handle large farms and do not work well for early disease detection.

Recent improvements in deep learning (DL) now offer effective replacements for human eye disease detection. It is worth noting that CNNs have achieved good results by recognizing textures and local features from leaf images, helping with proper plant disease classification (Pacal and Işık 2025; Setiawan et al. 2022). However, CNNs cannot efficiently represent relationships that take place over a wide area in the image. Because of these concerns, scientists now consider vision transformers (ViTs) a promising approach. ViTs use attention to each patch of an image to gather global information, which aids in seeing the entire pattern of disease. At the same time, these techniques need significant labeled data and a lot of computing power, which can be challenging to find in areas where farming takes place.

Therefore, a hybrid architecture was developed by combining CNNs and ViTs for the classification of maize leaf diseases. CNN allows us to find close‐up details, while ViT extracts the bigger picture relationships in areas of the image that are far apart. A combination strategy attaches these matching attributes into just one representation and sends it to the dense regions for checking.

Our work's primary contributions can be summarized as follows:

1. A comprehensive pipeline of preprocessing operations was implemented, comprising enlarging the maize leaves images to 256 × 256 pixels, rotation, random flipping, and normalization. This guarantees the elimination of undesirable noise and the standardization of images for the model input.
2. This study proposes a hybrid architecture that combines ViT and CNN. The ViT component records the global contextual relationships between various input image patches, whereas the CNN is utilized to extract local features.
3. This work presents a novel concatenation technique, which combines data from the CNN and ViT into a single feature vector that is then processed through dense layers to obtain the final classification. This improves the model's capacity to recognize local and global picture patterns, resulting in more precise forecasts.

Tests on data from Mendeley and Kaggle, plus cross‐validation, showed that the proposed model achieves an average accuracy of 99.06%. The model was also evaluated using a separate dataset called the corn disease and severity (CD&S), and it reached a strong accuracy of 95.93%. These outcomes support the idea that the model can be used in a range of agricultural conditions and provide valuable insights into scaling it for precise implementation in agricultural systems. Tests on data from Mendeley and Kaggle, plus cross‐validation, showed that the proposed model achieves an average accuracy of 99.06%. The model was also evaluated using a separate dataset called the CD&S, and it reached a strong accuracy of 95.93%. These outcomes support the idea that the model can be used in a range of agricultural conditions and provide valuable insights for scaling it for precise implementation in agricultural systems.

The remainder of this paper is organized as follows: Section II presents a review of recent deep learning research and its applications in agriculture. Section III explains the proposed methodology, datasets, and model architecture. Section IV discusses the experimental results and comparative evaluation with baseline models. Finally, Section V concludes the study and outlines directions for future research.

## 2 Overview of the Literature

Scientific studies have extensively explored plant disease feature extraction, classification, and recognition using deep learning (DL), machine learning (ML), and image processing techniques. The accuracy and efficiency of maize leaf disease recognition systems have significantly improved with the integration of DL models. The following section categorizes existing research based on its primary methodological approach, including CNN‐based models, ViT‐based models, hybrid architectures, and traditional ML techniques.

CNNs have remained the principal approach for maize disease classification due to their strong capability in detecting spatially meaningful patterns. Numerous studies have employed various CNN architectures to enhance accuracy in disease classification tasks. In addition to image‐based plant health assessment, CNNs have been applied to plant canopy reflectance analysis using multispectral data (He et al. 2025), aligning with the trend of applying CNNs and ViTs for agricultural imaging tasks (Kundu et al. 2022). In (Amin et al. 2022), EfficientNetB0 and DenseNet121 were used to extract deep features before integration, achieving 98.56% accuracy—outperforming ResNet152 (98.37%) and InceptionV3 (96.26%). The study in (Hu et al. 2020) employed transfer learning with a modified Google LeNet on a subset of PlantVillage, reaching an accuracy of 97.6%. In (da Rocha et al. 2020), AlexNet, ResNet50, and SqueezeNet were evaluated using Bayesian optimization to tune hyperparameters such as batch size and learning rate, achieving 97% accuracy with stratified k‐fold cross‐validation. The authors in (Pushpa et al. 2021) proposed a CNN‐based AlexNet model for detecting agricultural leaf diseases. Compared to VGG‐16 and LeNet‐5, AlexNet achieved 96.76% accuracy on a dataset of 7070 images of healthy and diseased leaves for tomato, maize, and rice, sourced from PlantVillage. Reference (Tariq et al. 2024) introduced a maize leaf disease classification method using VGG16 with layer‐wise relevance propagation (LRP), trained on a dataset of 4188 images comprising healthy and diseased samples. Their model achieved an accuracy of 94.67% and precision of 92.91%. The studies in (Pratama and Pristyanto 2023; Arjunagi and Patil 2023) evaluated the performance of AlexNet, LeNet, and MobileNet on a Kaggle maize leaf dataset. Among these, MobileNet showed promising results with 83.37% accuracy and precision and a g‐mean of 0.8298, as validated by agricultural experts (Gerber et al. 2021).

In (Xu et al. 2021), maize seeds were classified into five varieties using images of 8080 seeds through ML techniques, where SVM outperformed other models with 96.46% accuracy. The study in (Fraiwan et al. 2022) utilized deep transfer learning for classifying healthy plants and three maize diseases, testing multiple data‐splitting scenarios over 10 runs to reduce selection bias, achieving an average accuracy of 98.6%. Reference (Prakash and Kirubakaran 2023) applied ResNet152 for feature extraction, outperforming other models with 98.54% classification accuracy.

In (Sharma et al. 2023), a CNN based on ResNet34 was trained using the PlantVillage dataset to classify maize diseases into four classes, achieving an F1‐score of 0.93, precision of 94%, and mean accuracy of 97.6%. These results highlight the feasibility of decision‐support tools for farmers and plant pathologists. In (Verma et al. 2024), a lightweight CNN model was proposed for disease detection in maize, rice, and wheat using multi‐scale convolutional layers. It achieved an overall accuracy of 84.4% with only 387,340 parameters and high class‐wise accuracy: 99.74% for maize, 82.67% for rice, and 97.5% for wheat. The ICS‐ResNet model, a lightweight architecture based on ResNet50, achieved 98.87% accuracy with 54.88% fewer computations and 69.21% fewer parameters compared to prior models (Ji et al. 2024). In (Wu 2024), ResNet18 with data augmentation and preprocessing achieved 98% accuracy, 95% precision, 95% recall, and 95% F1‐score, confirming its effectiveness in crop disease diagnosis.

Reference (Haque et al. 2022) proposed a fine‐tuned InceptionV3 model that achieved 95.99% accuracy. In (Theerthagiri et al. 2024), the authors classified three maize leaf diseases using VGG16, ResNet34, ResNet50, and SqueezeNet trained on a balanced Kaggle dataset, implementing techniques like SMOTE to counter overfitting. A hybrid CNN‐ViT model was also proposed, combining local and global feature extraction mechanisms for robust maize leaf disease detection. This model was tested on two publicly available datasets to ensure its real‐world applicability. Table 1 presents a comprehensive summary of the literature reviewed.

**TABLE 1:** Description of merged dataset images.

Dataset	Healthy	NLB	GLS	CR
Corn/Maize Leaf Disease Dataset‐1	1162	1146	574	1306
Maize Leaf Disease Dataset‐2	1162	985	513	1192
Total	2324	2131	1087	2498

A recent study (Pacal 2024) proposed an advanced vision transformer model based on the multi‐axis vision transformer (MaxViT) for maize leaf disease classification. The model was enhanced by integrating a Squeeze‐and‐Excitation (SE) block in the stem and replacing the standard MLP with a GRN‐based MLP from ConvNeXtV2 to improve both accuracy and inference speed. The researchers combined the PlantVillage, PlantDoc, and CD&S datasets to create the largest known maize disease dataset and conducted a comprehensive comparison involving over 28 CNNs and 36 transformer models. A recent study employed advanced ViT models, alongside CNN architectures like VGG, ResNet, DenseNet, and Xception, for corn leaf disease detection. The models were trained on both the PlantVillage and the novel CD&S datasets, using techniques such as transfer learning, data augmentation, and an adaptive soft voting ensemble. The approach achieved 100% accuracy on the CD&S test set and 99.83% on the PlantVillage dataset, demonstrating that ViT‐based models can outperform traditional CNNs in crop disease diagnosis. This study focuses on the classification of four distinct maize disease classes.

## 3 Proposed Methodology

**FIGURE 1:**
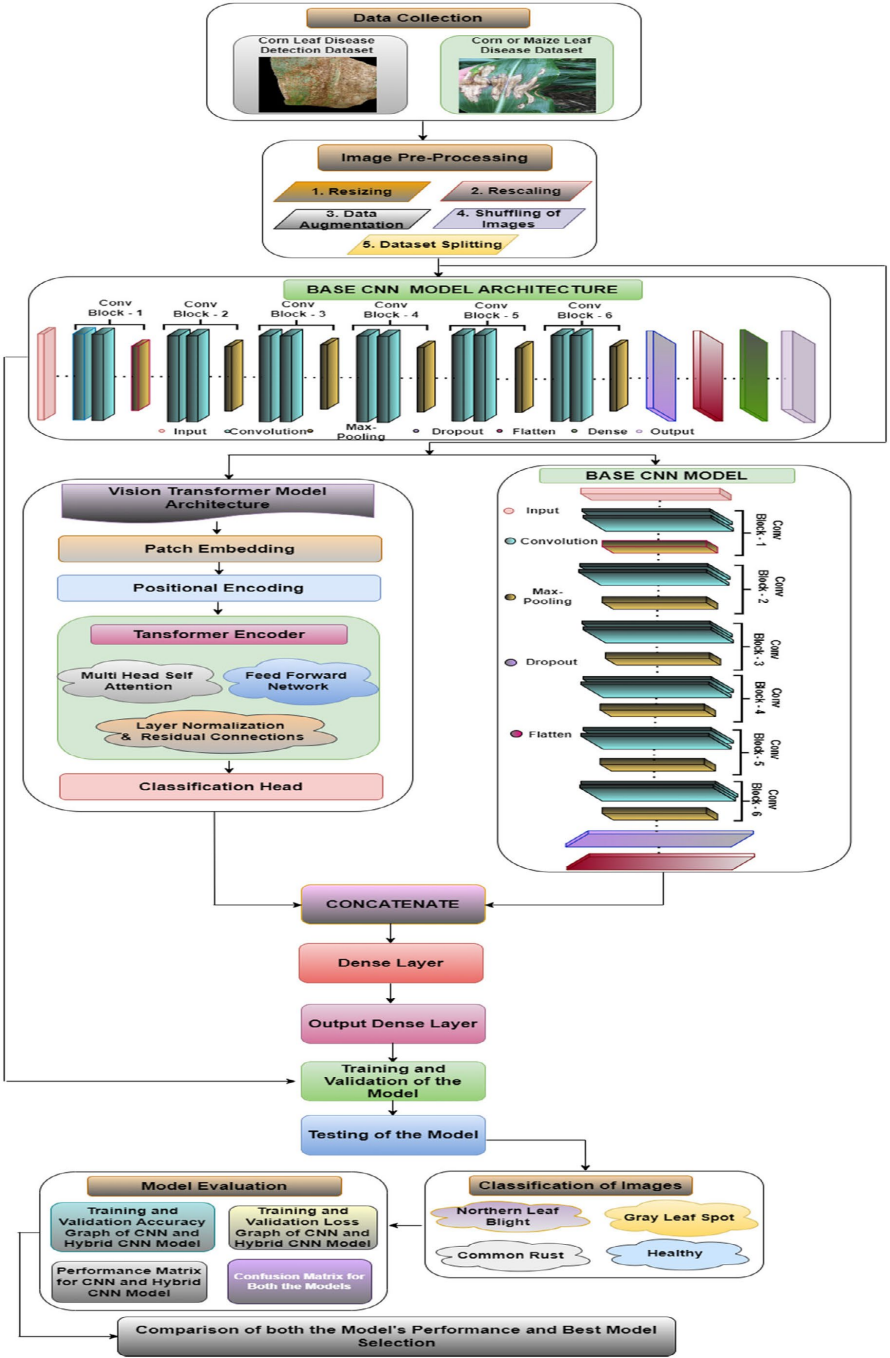
Proposed methodology.

This section discusses the proposed framework for the categorization of this maize plant disease. Figure 1 displays the proposed methodology for this study. Data collection stands as the initial step that is shown in Figure 1. In this study, two datasets, the Maize or Maize Leaf Disease Detection dataset from Kaggle and the Maize Leaf Disease Detection dataset from Mendeley, were used for model training and testing. These datasets contain the same four classes for maize leaf diseases, which were considered in this study. After the data collection step, the images are preprocessed to clean the data so that unwanted noise can be removed from the images and the data can be transformed per the model requirements. In preprocessing, the images are resized to a uniform size of 256 × 256 pixel resolution. The images are subsequently normalized in the range of (0, 1). Data augmentation is applied to the images in which the images are randomly flipped and rotated. This step increases the number of images so that the imbalance class issue can be resolved. This step is further followed by the shuffling of images so that the same pattern images are not repeated. The images are then split into three sets at a ratio of 8:1:1 for training, testing and validation. After these preprocessing steps, the images are ready to be fed into the model for training. The proposed methodology is divided into two parts. First, to identify local characteristics in the images, a CNN model is built. To categorize the input maize leaf images into four distinct disease categories, the model design includes convolutional layers as well as max pooling layers and dropout layers alongside fully connected dense layers. The next step involves training and assessing the CNN model on the preprocessed images. Furthermore, the model is evaluated, and different performance measures are recorded.

Step 3 of the study involves creating a CNN + ViT hybrid model. The CNN component is retained for local feature extraction, whereas the ViT framework is added to collect global contextual associations inside the image patches. The outputs of the CNN and ViT are subsequently concatenated and sent through dense layers for classification. A single feature vector that combines local and global features is created by concatenating the output feature maps from the CNN and ViT. To discover more complex patterns, the concatenated characteristics are processed through fully connected layers. With each leaf image, the final dense layer predicts the maize disease category on the basis of the classification findings. The next step involves using the same datasets to train and assess the hybrid CNN + ViT model. Both models are tested on the test dataset. The hybrid model and the original CNN are compared via the same performance metrics. Finally, a comparison of the CNN and hybrid models' outputs is made, emphasizing the gains in accuracy and overall classification performance that the hybrid architecture has brought about. In the final analysis, the results are summarized, and the advantages of the hybrid model for disease classification tasks are highlighted.

### 3.1 Input Dataset

In this study, two datasets, corn/maize leaf disease dataset‐1 and maize leaf disease detection dataset‐2, were combined for the classification of maize disease images. The Mendeley maize leaf disease dataset, collected under real field conditions in Ghana, and the Kaggle dataset derived from the PlantVillage repository, were captured in controlled lab‐like settings. While both datasets contain the same four class labels, Blight, Common Rust, Gray Leaf Spot, and Healthy, they differ significantly in visual characteristics, including lighting, background context, camera angle, and noise. The Mendeley dataset introduces natural variance found in real‐world agricultural environments, while the Kaggle dataset provides cleaner, high‐contrast imagery. This heterogeneity enriches the training set but also raises the potential for domain shift and dataset‐induced bias. To mitigate this, we applied consistent pre‐processing: all images were resized to a unified resolution (e.g., 256 × 256), normalized in pixel intensity, and augmented through random flipping, rotation, zoom, and brightness adjustment. Additionally, stratified shuffling ensured proportional class and domain representation across training, validation, and test splits. These measures help the model focus on disease‐specific visual cues rather than dataset‐specific artifacts, improving generalizability in real‐world deployments.

#### 3.1.1 Corn/Maize Leaf Disease Dataset‐1

**FIGURE 2:**
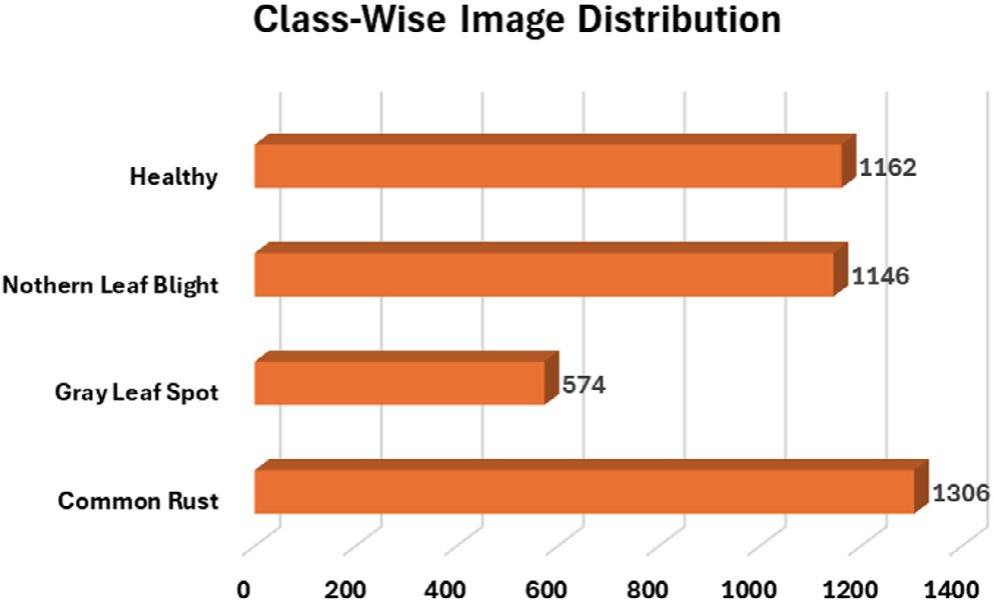
Class‐wise distribution of images.

**FIGURE 3:**
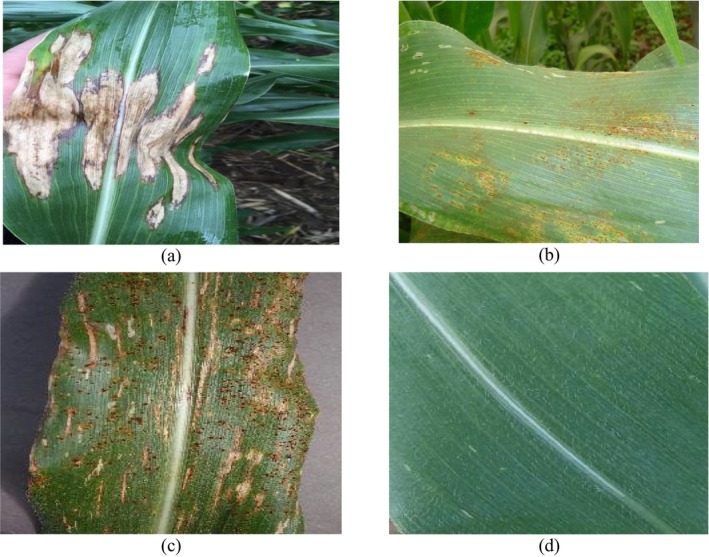
Sample images: (a) NLB, (b) CR, (c) GLS, and (d) healthy class (https://www.kaggle.com/datasets/smaranjitghose/corn‐or‐maize‐leaf‐disease‐dataset).

The publicly accessible corn or maize leaf disease Dataset, which can be found on Kaggle (https://www.kaggle.com/datasets/smaranjitghose/corn‐or‐maize‐leaf‐disease‐dataset), enables the classification of maize leaf diseases. The dataset contains digitally processed maize leaf image files classified into four disease categories which include three illnesses and one category of healthy leaves. The dataset serves as an optimal resource for DL model training purposes devoted to plant disease detection and classification systems. Two agricultural databases PlantVillage and PlantDoc delivered expert‐labeled images of agricultural subjects amounting to thousands of images. The data collection process excluded substandard images while retaining only those with distinct disease symptoms showing in high‐definition resolution. To maintain data consistency, the dataset went through expert verification which confirmed the correct disease type labels. This dataset has 4188 maize plant disease images. It consists of four classes of images: CR, GLS, NLB, and healthy maize plant images. In the dataset, the images are labeled carefully according to their class and disease symptoms. The CR class is labeled Class 0, the GLS is labeled Class 1, the NLB is labeled Class 2, and the healthy category is assigned a Class 3 label. Figure 2 shows the details of the images classwise. Figure 3 displays the sample images of the dataset.

#### 3.1.2 Maize Leaf Disease Dataset‐2

**FIGURE 4:**
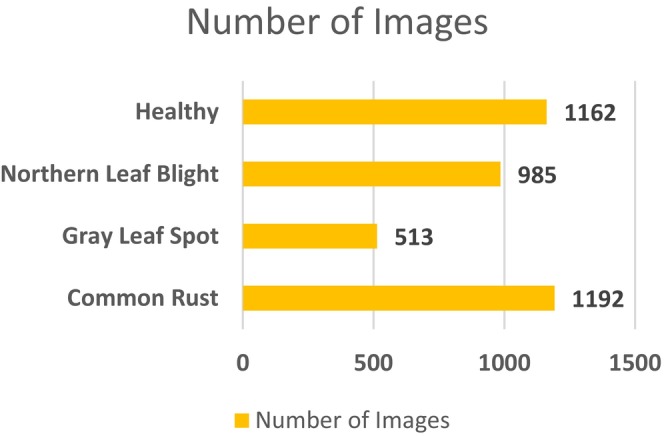
Distribution of image classes.

**FIGURE 5:**
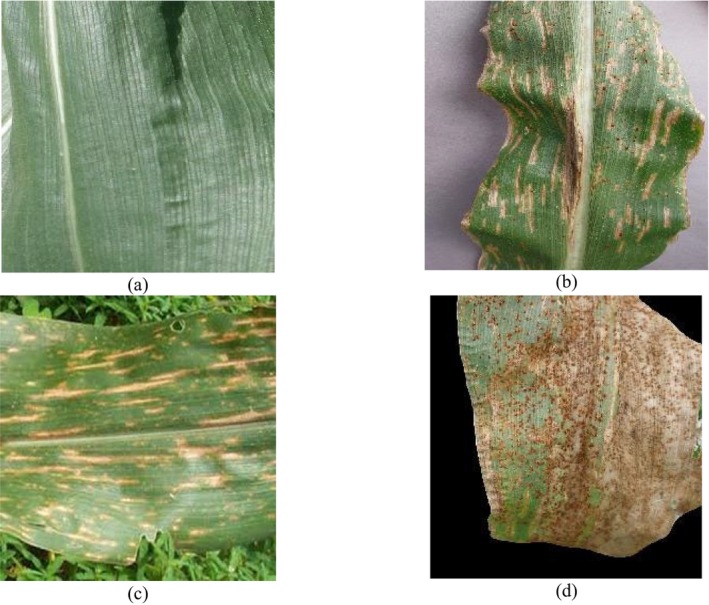
Sample images: (a) healthy, (b) NLB, (c) GLS, and (d) CR class (Arun Pandian 2019).

The other dataset that is employed to conduct this categorization task is the maize leaf disease detection dataset (Arun Pandian 2019), which is available publicly on Mendeley. This dataset contains images for 39 different classes of plant diseases. Among these, images of maize plant diseases have been utilized to conduct this maize disease detection task. This dataset consists of 3852 maize images for four classes. High‐resolution cameras operated directly from Ghanaian farms delivered images that presented actual field conditions through diverse examples. A wide range of environmental conditions and backgrounds from white to dark, illuminated to natural farm environments, were used to strengthen the dataset. Plant virologists who are experts performed a thorough examination of all images to establish proper labels along with anonymization procedures for accurate classification. The Mendeley dataset brings diverse real‐world variance in lighting, leaf occlusion, and background noise, making it ideal for testing model generalization across variable conditions. Figure 4 provides the information on the class‐wise images. Figure 5 displays the sample images for the four classes of maize diseases.

#### 3.1.3 Corn Disease and Severity (CD&S) Dataset

To evaluate the generalization ability of the proposed Hybrid CNN‐ViT model beyond curated datasets, we employed the CD&S dataset (Ahmad et al. 2021) as an external benchmark. This dataset includes a total of 4455 images, consisting of both raw and augmented samples. For the purpose of this study, only the 1597 raw images were used for image‐level classification. These images were captured in real‐world field conditions at Purdue University's Agronomy Center for Research and Education (ACRE) in West Lafayette, Indiana, during July 2020. A 12‐megapixel iPhone 11 Pro was used to collect images at a resolution of 3000 × 3000 pixels, maintaining a 1:1 aspect ratio that is well‐suited for deep learning applications. The dataset comprises three major maize diseases: northern leaf blight (NLB) with 511 images, gray leaf spot (GLS) with 524 images, and northern leaf spot (NLS) with 562 images. Overall, the CD&S dataset comprises 4455 images, including 2112 raw images (classification and severity estimation) and 2343 augmented images, making it a robust benchmark for training and evaluating deep learning models in realistic agricultural settings.

### 3.2 Preprocessing

To conduct this categorization study, both Dataset‐1 and Dataset‐2 were merged. Table 1 lists the details of the class‐wise images after these two datasets were merged. The table shows that for the healthy class, there are 2324 images; for the NLB, there are 2131 images; for the GLS, there are 1087 images; and for the CR class, there are 2498 images. For this study, a total of 8040 images were employed for this maize disease classification task.

**FIGURE 6:**
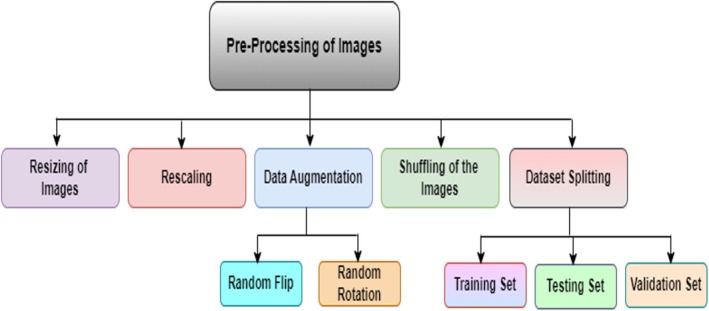
Preprocessing steps.

The images have been collected from various sources; thus, these images can be of different sizes and may contain noise, which can create a problem during the training phase of the model. Figure 6 shows the preprocessing process flow for the preparation of the dataset. The figure shows that this process includes several steps. These are discussed below along with the mathematical derivation followed during the implementation of these steps.

#### 3.2.1 Resizing

This process starts with the resizing of images to increase the uniformity of the dataset. The images are first resized to a resolution of 256 × 256 to ensure consistent spatial dimensions across the dataset and to maintain a balance between retaining fine‐grained disease features and enabling efficient computation during training and transformer‐based patch embedding. Through interpolation, the resizing procedure modifies the overall size of the input image. Supposing linear interpolation, the following bilinear interpolation formula can be used to represent the resizing action mathematically:

(1) $$I^{\prime }\left(a^{\prime },b^{\prime }\right)=I\;\left(\;\frac{a^{\prime }}{h}\times H,\frac{b^{\prime }}{w}\times W\;\right)$$

where, *I* (*a*, *b*) is the original image, *I*′(*a*', *b*') is the resized image, *H* × *W* is the dimension of the original image, and *h* × *w* is the size of the target image (256 × 256).

#### 3.2.2 Rescaling

The images are normalized in this step. This step improves convergence, stability, and efficiency by transforming the pixel values into a range more appropriate for the training of neural networks. In mathematical terms, the rescaling procedure for each pixel p in the original image is given by

(2) $$p^{\prime }=p/255$$

where $p^{\prime }$ is the rescaled pixel value.

**FIGURE 7:**
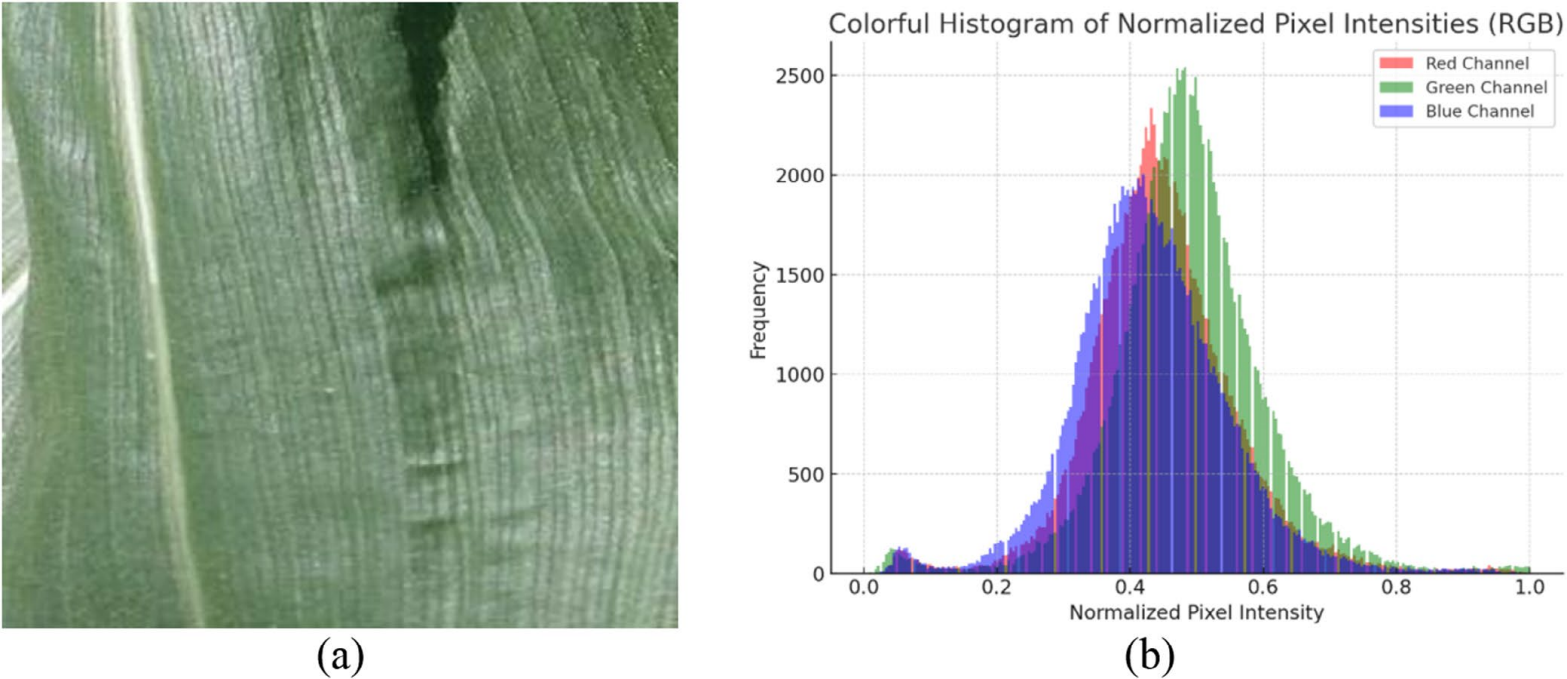
(a) Normalized image and (b) histogram of the normalized image.

Figure 7 shows a histogram representation of the normalized image pixel intensities. After the image was normalized, the intensity of each pixel was between 0 and 1. For improved visual aids, red, green, and blue are used to symbolize the various channels.

#### 3.2.3 Data Augmentation

Figures 2 and 3 shows that both datasets are imbalanced, as the GLS class has fewer images than the other classes do. Therefore, to solve this issue, data augmentation is applied to the dataset. A method called “data augmentation” is used; especially in image‐based applications, this technique enhances data quantity and diversity through synthetic manipulation of available dataset information. This helps models generalize better by providing diverse representations of the same objects. These adjustments improve the model's capacity for generalization while preventing overfitting. In this study, two image transformations, random flipping and random rotation, were performed on the images. While performing random flipping, the images are randomly flipped horizontally and vertically. These transformations were chosen to simulate real‐world variances in leaf orientation and capture conditions, thereby enhancing the model's generalization capability. In mathematical terms, flipping an image horizontally is represented by reversing the pixel coordinates along the *x*‐axis:

(3) $$I^{h}\left(a,b\right)=I\left(W-1-a,b\right)$$

where *I (a, b)* is the actual image, $I^{h}\left(a,b\right)$ is the horizontal flipped image, and *W* is the width of the image.

Similarly, a vertical flip can be shown by flipping the pixel coordinates along the y‐axis:

(4) $$I^{v}\left(a,b\right)=I\left(a,H-1-b\right)$$

Here, *H* is the height of the image, and $I^{v}\left(a,b\right)$ is the vertically flipped image.

**FIGURE 8:**
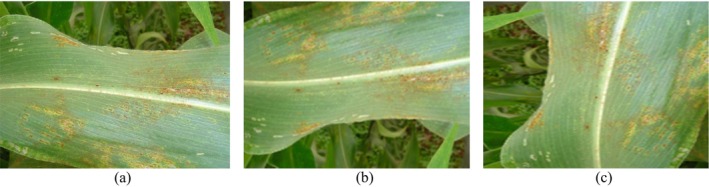
Data augmentation; (a) original image; (b) vertical flipping; and (c) rotating the image by 90°.

The other transformation applied to the image is random rotation. This variation in orientation helps the model learn the object's features despite the changes in the angle. It enhances the model's performance by providing it with the ability to recognize an object from different angles, which is crucial for tasks like object detection or classification, especially when the object is seen in various orientations in real‐world scenarios. The images are randomly rotated by a factor of 0.2. This indicates that its rotation angle is between [−0.2 × π, 0.2 × π] radians. Figure 8 shows the transformations performed on the image.

#### 3.2.4 Shuffling of Images

After data augmentation, the images are shuffled to reduce the chance of bias toward any class. The Fisher‐Yates shuffling algorithm was used in this study to implement this shuffling step. Using a series of random image flips over a dataset, the Fisher‐Yates shuffle creates a fresh randomized sequence. This ensures that there is no intrinsic order in the data, which improves generalization and prevents overfitting.

#### 3.2.5 Dataset Splitting

**FIGURE 9:**
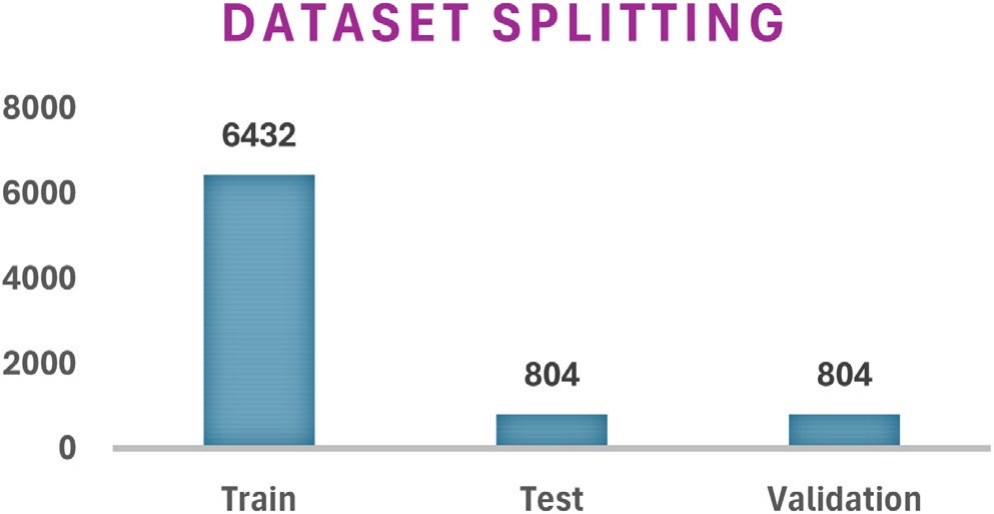
Splitting of dataset.

The dataset splitting includes dividing it into three distinct parts which serve training and testing and validation functions. Both datasets are split at a ratio of 8:1:1. The split ratio demonstrates how the images are distributed with 80% going to training and 20% being equally distributed for training and validation processes. The dataset contains 8040 images following the combination of images from both previous datasets. Figure 9 shows the details of the setwise images after dataset splitting.

### 3.3 Base Convolutional Neural Network Model

**FIGURE 10:**
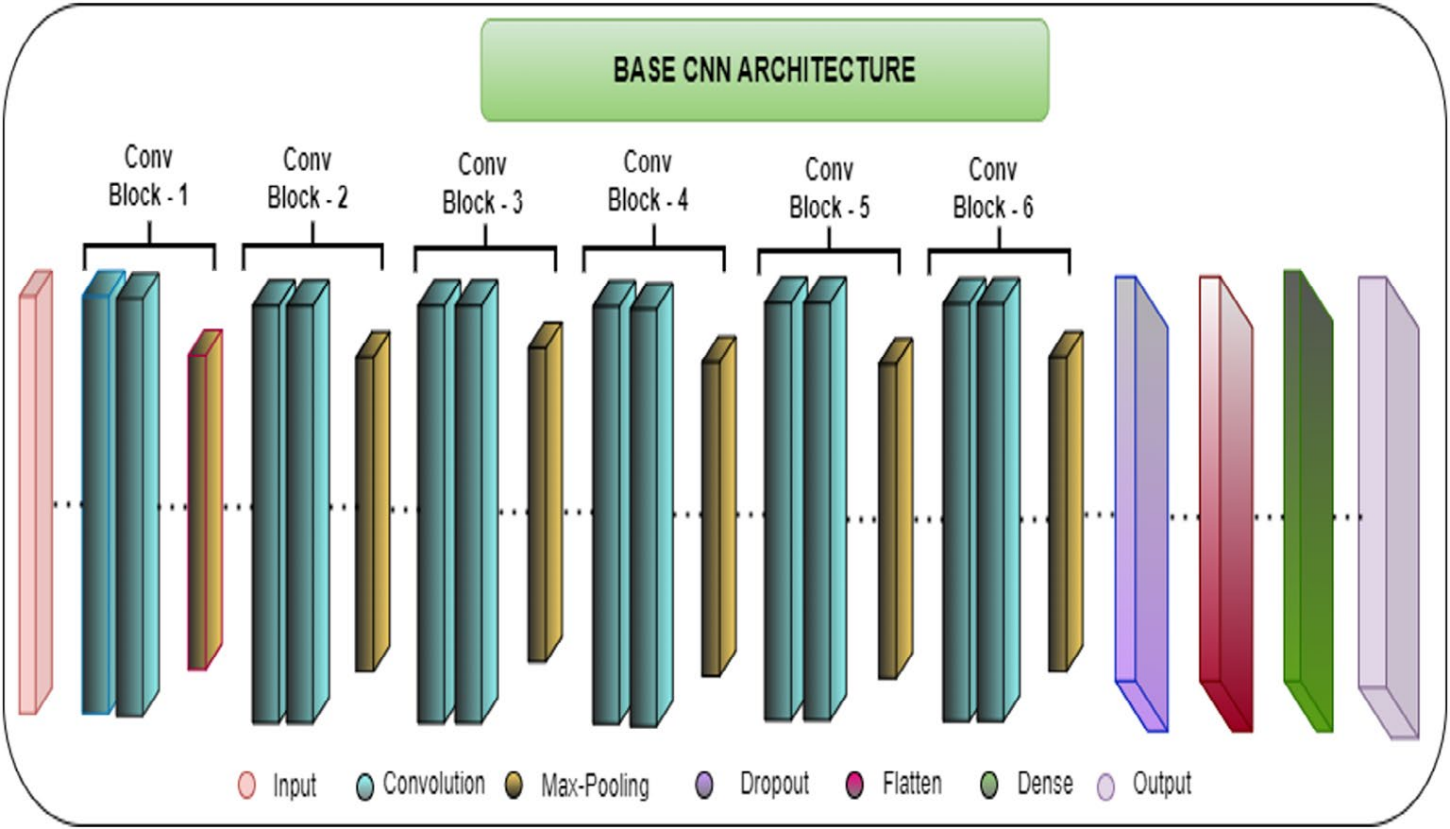
Base CNN model architecture.

To conduct this study, first, a base CNN model is designed. Six convolution blocks and six max pooling layers are used to design this method. Apart from this, one dropout layer, one flattening layer, and two dense layers are used. The last dense layer works as an output layer, which categorizes the maize leaf images into four categories based on their features. Every layer has a unique work that helps in the feature extraction process. Figure 10 shows the basic CNN model architecture.

Table 2 gives a model summary of the basic CNN model. The details of every layer along with their work are discussed below.

- Convolutional layer: This layer helps in the feature extraction process. Convolution filters are capable of capturing the smaller elements in an image, such as maize, textures, and borders. Convolution layers begin by extracting simple patterns (textures and edges) from the input images and progress to more intricate characteristics (shapes and objects). By removing negative values and keeping only positive activations, applying ReLU activation with a convolutional layer aids in the network's learning of nonlinear patterns (Sun and Huo 2025). The table shows that the Conv2D first layer applies 32 convolution filters or kernels of size 3 × 3 over the input image. A convolution filter, sometimes known as a kernel, is a matrix that moves across the whole input image and multiplies the values of the image patch beneath it element‐by‐element. To obtain a single output value, which is referred to as a feature, the results are added together. Furthermore, the table shows that the convolution layers apply another set of 64 filters of size 3 × 3. Convolution is performed with the formula stated in equation (5):

(5) $$y\left(i,j\right)=\sum_{m=1}^{3}\sum_{n=1}^{3}W\left(m,n\right).X\left(i+m,j+n\right)+b$$

where, *X* is the input image or feature map. *W* is the convolutional filter or kernel. (*i*, *j*) are the positions of the filter on the input image. Location within the 3 × 3 filter, traversing over its rows and columns, is indicated by m and n. b is the bias added after the completion of the convolution process.

- Max‐pooling layer: Max‐pooling layers reduce the spatial size of the network while maintaining critical information by downsampling the feature maps. This increases the computational efficiency of the network. There are minor sections inside the input image. The max pooling operation chooses the highest quantity across each specific region. The input dimension reduces because this method retains only maximum values for each specific zone. The max pooling layer is calculated with the formula stated in equation (6):

(6) $$y\;\left(i,j\right)=\max\;\left(X\;\left(m,n\right)\right)\;\text{for}\;\mathrm{m\epsilon}\;\left[i,i+k-1\right],\;\mathrm{n\epsilon}\;\left[j,j+k-1\right]$$

where, *X* (*m*, *n*) is given as pixel values in the *k* × *k* region of the input feature map. *y* (*i*, *j*) is the output value after max pooling at position (*i*, *j*), which is the maximum value in the *k* × *k* region.

- Dropout layer: To prevent overfitting issues, the dropout layer in neural networks serves as a regularization technique that momentarily turns off neurons for training by random selection (Rani et al. 2025). This method helps the model learn more resilient and universal properties by avoiding overreliance on any one neuron. While the remaining neurons support the network's forward pass and backpropagation, a portion of the neurons, in this case, 50% are randomly “dropped out,” which means that their outputs are set to zero during training. A neuron's post‐dropout output can be calculated via equation (7):

(7) $$\hat{z}=z.M$$

where, *z* represents the initial output. *M* is a random binary mask that, depending on the dropout rate (*p*), either deactivates the neuron with a probability of *p* or keeps it active with a probability of 1−𝑝.

- Flatten layer: Through their filtering operations and pooling stages CNNs produce their final output as three‐dimensional tensors using three measurements of pixel dimensions and input channels. Dense or fully connected layers accept a 1D vector as their input compatibility. Fully connected densities accept a one‐dimensional vector as their input through this layer's dimensional conversion capabilities. Mathematically, flattening transforms a tensor, which is expressed in Equation (8):

(8) $$X\;\epsilon \;R^{h\;x\;w\;x\;c}$$

Here, *h* is the height and *w* is given as the width of the image feature map, and *c* is the number of channels in the feature map.

**TABLE 2:** Base CNN model parameters.

Layer	Output shape	Parameters
Input (Sequential)	(32, 256, 256, 3, 0)	0
Conv2D – 1	(32, 254, 254, 32)	896
Max‐Pool2D – 1	(32, 127, 127, 32)	0
Conv2D – 2	(32, 125, 125, 64)	18,496
Max‐Pool2D – 2	(32, 62, 62, 64)	0
Conv2D – 3	(32, 60, 60, 64)	36,928
Max‐Pool2D – 3	(32, 30, 30, 64)	0
Conv2D – 4	(32, 28, 28, 64)	36,928
Max‐Pool2D – 4	(32, 14, 14, 64)	0
Conv2D – 5	(32, 12, 12, 64)	36,928
Max‐Pool2D – 5	(32, 6, 6, 64)	0
Conv2D – 6	(32, 4, 4, 64)	36,928
Max‐Pool2D – 6	(32, 2, 2, 64)	0
Dropout (0.5)	(32, 2, 2, 64)	0
Flatten	(32, 256)	0
Dense	(32, 64)	16,448
Dense (Output)	(32, 4)	260

*Note:* Total Parameters = 183,812. Trainable Parameters = 183,812. Non‐Trainable Parameters = 0.

Flatten transforms it into a 1D vector Y of size stated in equation (9):

(9) $$Y\;\epsilon \;R^{h\;x\;w\;x\;c}$$

- Dense layer: Fully linked or dense layers form the core feature of neural networks by connecting every neuron to all neurons in the next higher layer. In many neural network topologies, particularly convolutional or pooling layers in CNNs, this layer is in charge of acquiring representations at the highest level and making the final evaluation. The following formula is used to calculate the output of a dense layer with biases b for a given input vector X and weight matrix W:

(10) Z=W.X+b

where, *X* is the input vector, which is flattened from previous layers. The weight matrix, or *W*, is made up of weights that each indicate how strongly input and output neurons are connected. The bias vector, or *b*, aids in shifting the activation function. *Z* is given as the output provided by the layer before activation is applied.

- Output layer: The output layer is the final layer of a neural network and is in charge of making predictions. The specific objective of classification or regression dictates the shape of the output layer. The output layer uses a Sigmoid or SoftMax activation function to produce probabilities for each class in classification tasks. The multi‐class classification in this study was conducted via the SoftMax activation function. The SoftMax activation function converts the raw scores (or logits into probabilities for each class). The SoftMax function for class i is given by equation (11):

(11) $$P\left(y_{i}\right)=\frac{e^{Z_{i}}}{\sum_{j=1}^{n}e^{Z_{i}}}$$

where, $P\;\left(y_{i}\right)$ is given as the predicted probability for class i. $Z_{i}$ is the output for class I before the application of the SoftMax activation function. *n* is the total number of classes.

The SoftMax function is appropriate for classification jobs since it guarantees that all output values are in the range [0, 1] and that the total probability for all classes equals 1 (Rani et al. 2025). Table 2 lists the trainable and nontrainable parameters for the base CNN model.

### 3.4 Vision Transformer Model

**FIGURE 11:**
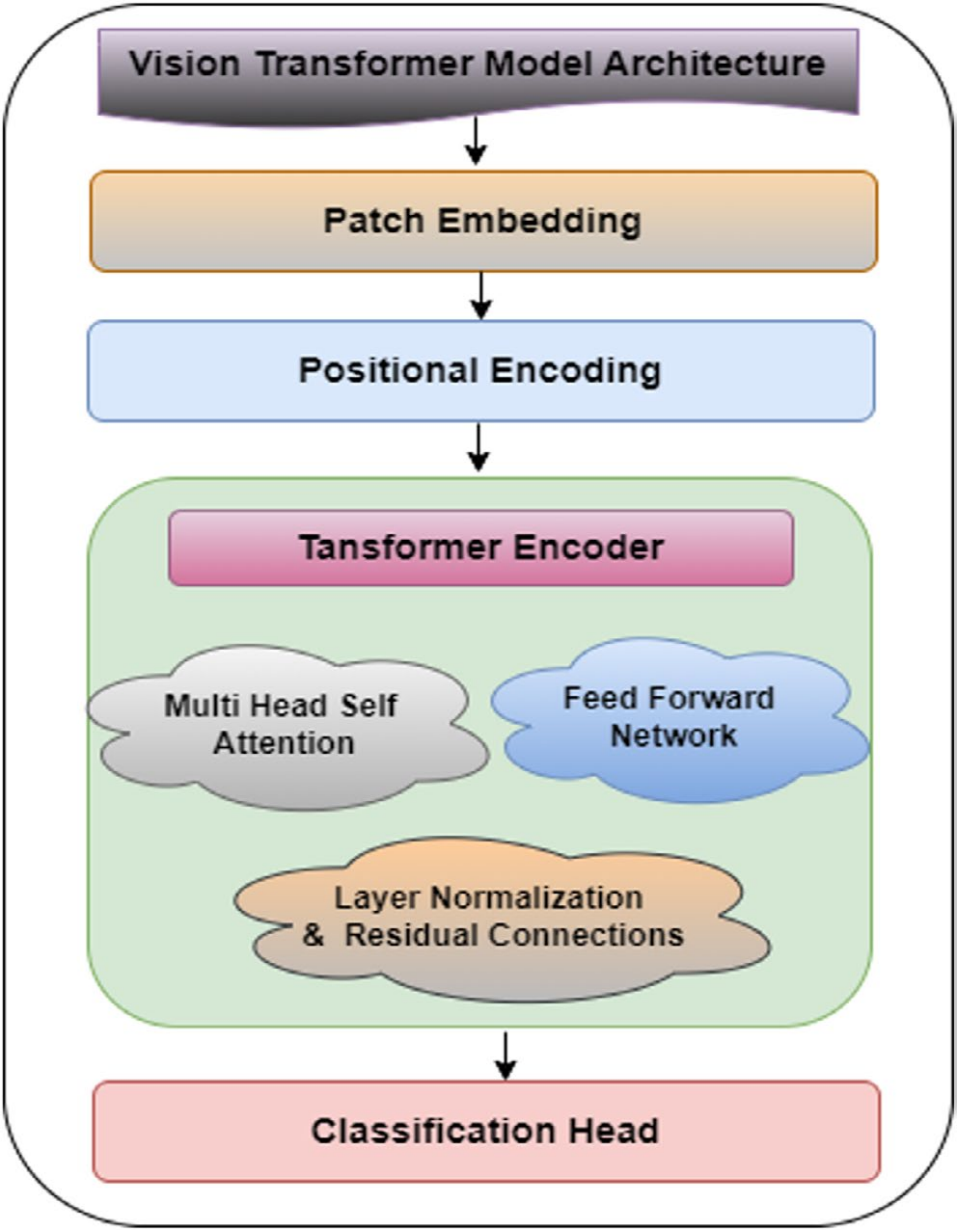
Architecture of vision transformer.

The ViT model framework represents a novel approach to the image categorization task that employs a self‐attention mechanism. Unlike CNNs, ViTs do not use convolution process operations for feature extraction. They view the images as a sequence of patches that are similar to words in a sentence (Pacal et al. 2024). This model outperforms CNN‐based models in the image classification task. This architecture comprises patch embedding, positional encoding, multihead self‐attention layers and a classification head. Figure 11 shows the architecture of the ViT model. The figure shows that the ViT model consists of various blocks, which are discussed below:

- Patch embedding: This is the process of converting an input image into a sequence of smaller, non‐overlapping patches and then representing each patch as a fixed‐dimensional vector. The image is represented in the form of height H, width W, and a color channel; if it is an RGB image, then, 3 images are taken. Each patch is linearly projected onto a lower‐dimensional embedding space after being flattened. Patch size *P* × P. Number of Patches.

(12) $$N=H\times Y/P^{2}$$

The patches are then reshaped into vectors of size *P* ^2^ × *C*.

- Positional encoding: The incorporation of spatial information occurs through the addition of positional encodings to the overall patch embedding structure. Self‐attention layers consist of permutation‐invariance which lacks built‐in memory to keep track of patch position within the image framework. Thus these mandatory encodings ensure essential positional retention.

(13) $$z_{0}^{\prime }=z_{0}+E_{\mathrm{pos}}$$

The positional encodings $E_{\mathrm{pos}}$ are fixed or learned embeddings that correspond to the position of each patch in the sequence.

- Transformer encoder: The transformer encoder comprises multiple layers with a multi‐head self‐attention (MHSA) mechanism and a feed‐forward network (FFN) as successive parts. Each transformer encoder layer depends on L‐stacked instances of the identical structure.
  ○ Multi‐head self‐attention (MHSA): Self‐attention computes pairwise attention scores between patches. Each head in multi‐head attention computes scaled dot‐product attention, as stated in equation (14):

(14) $$\text{Attention}\left(Q,K,V\right)\;\text{softmax}\left(\;\frac{QK^{T}}{\sqrt{d_{k}}}\;\right)V$$

where *Q*, *K*, and *V* represent the query, key, and value matrices, respectively, and d_k_ is the dimension of the keys. Multihead attention allows the model to jointly address information from different representation subspaces at different positions.

○ Feed‐forward network (FFN): After the attention mechanism, the output passes through a two‐layer fully connected feedforward network with a GELU activation function, which is given by equation (15):

(15) $$\mathrm{FFN}\left(x\right)=\text{GELU}\;\left(xW_{1}\;b_{1}\right)W_{2}b_{2}$$

Layer normalization and residual connections: layer normalization and residual connections are applied both before and after the attention and FFN layers:

(16) $$x=\text{LayerNorm}\left(x+\text{MHSA}\left(x\right)\right)$$

(17) $$X=\text{LayerNorm}\;\left(x+\mathrm{FFN}\;\left(x\right)\right)$$

○ Classification head: A special learnable class token [CLS] is prepended to the sequence of patches. After the transformer blocks, the output corresponding to this token is passed through a classification head:

(18) $$y=\text{softmax}\left(W_{\mathrm{CLS}}\times z_{\mathrm{CLS}}\;\right)$$

### 3.5 Hybrid CNN‐Vision Transformer Model

**FIGURE 12:**
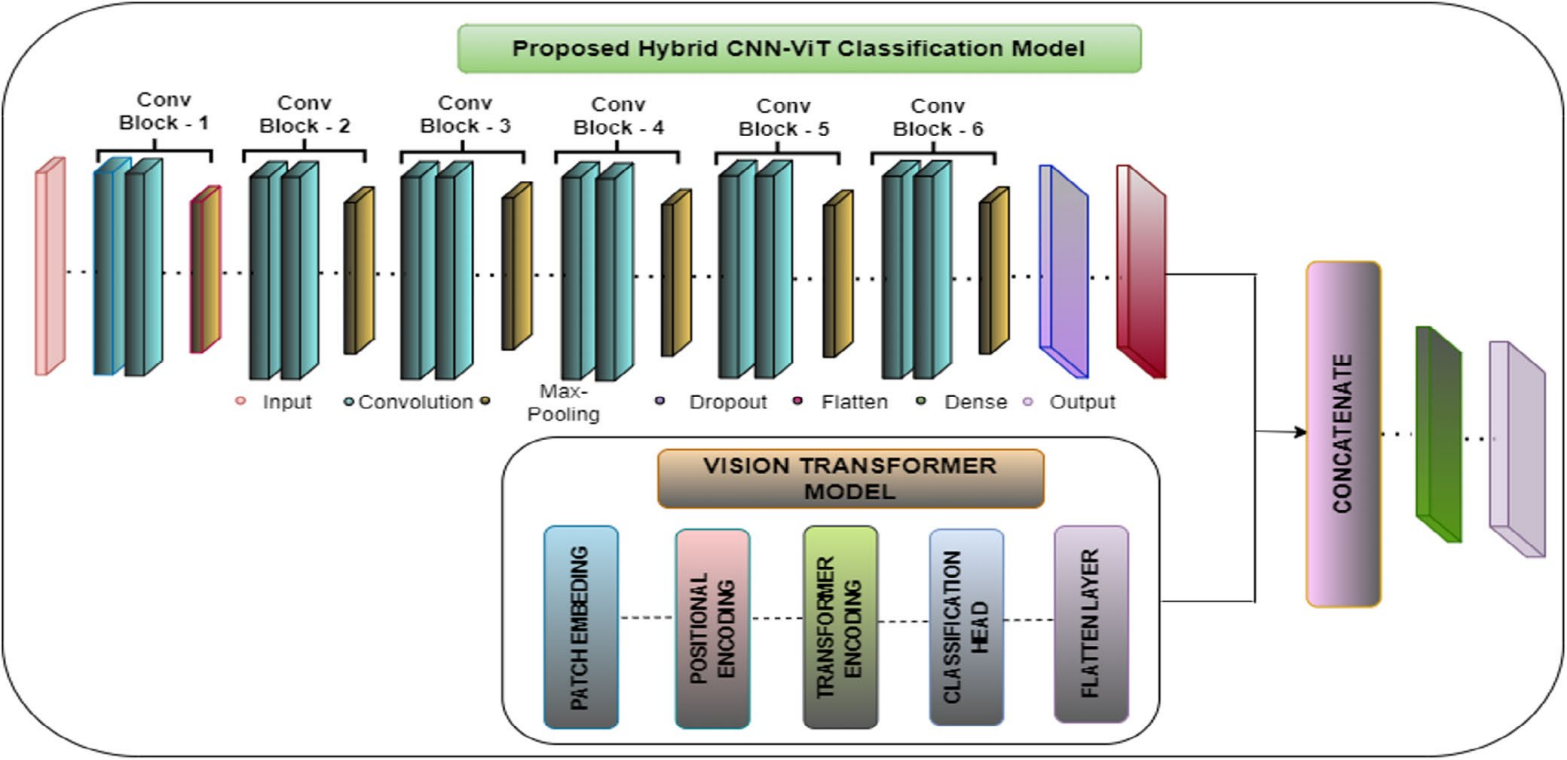
Proposed hybrid CNN architecture.

**FIGURE 13:**
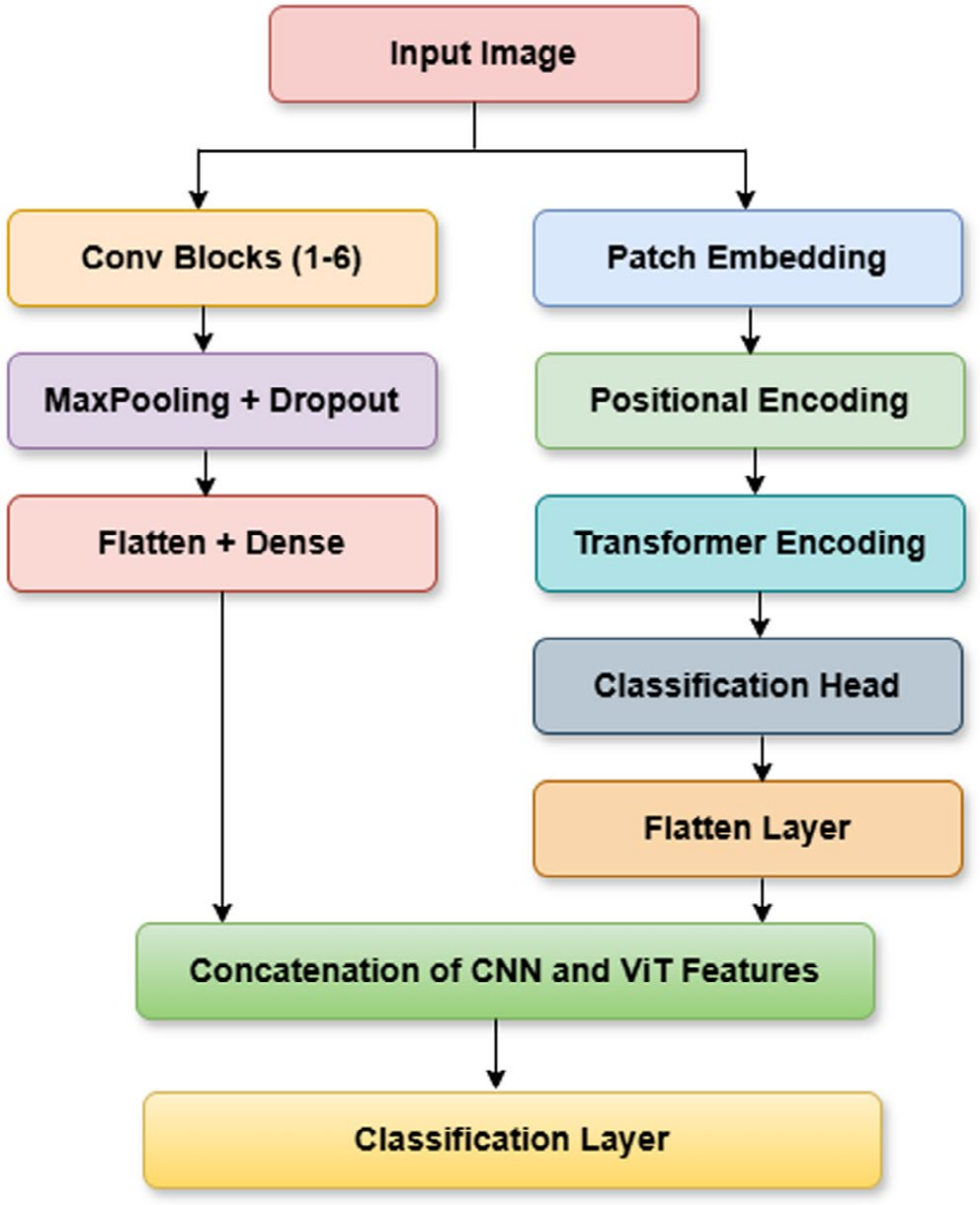
Workflow of the proposed hybrid CNN‐ViT Model.

The proposed hybrid CNN‐ViT classification model as shown in Figure 12 integrates the local feature extraction capabilities of CNN's with the global contextual understanding of ViTs to enhance maize leaf disease classification. From Figure 13 the working of this proposed Hybrid CNN‐ViT model can be seen from which the workflow can be analyzed easily. The CNN component comprises six convolutional blocks, where convolutional layers identify fine‐grained features, max‐pooling layers reduce dimensionality, and dropout layers help prevent overfitting. As the image progresses through these layers, the extracted features become increasingly abstract and informative. Once the final convolutional block processes the image, the extracted feature maps are flattened and passed through dense layers for further refinement.

At the same time, the ViT model processes the input image by dividing it into non‐overlapping patches, which are transformed into feature vectors. Positional encoding ensures the model retains the spatial relationships between these patches. These encoded representations are then fed into transformer layers equipped with self‐attention mechanisms, allowing the model to capture long‐range dependencies and global structural patterns. The outcome from ViT is a small feature vector that captures the whole image. The main strength of this architecture lies in combining CNN and ViT features, letting the model work with both local and global features. The CNN and ViT models each generate feature vectors that are then combined by the concatenation process to give the model a representation with both detailed and large‐picture information. It is then given to a fully connected dense layer with 64 neurons and the ReLU activation, and a dropout layer with a 0.5 dropout rate is added to stop overfitting. The last step is a dense layer with four neurons and a SoftMax activation function, which matches the number of maize disease classes. This improved set of features is fed through dense layers, ending with a classifying layer that puts maize leaves into one of four categories. After collecting local spatial and global context features from CNN and ViT, the outputs are combined into a single united feature vector. Following that, the mixed features go through a fully connected dense layer of 512 neurons with ReLU, helping the model transform the features in non‐linear ways. To avoid overfitting, some neurons are turned off randomly at a rate of 0.5 at each training phase. This is continued by adding another dense layer of 128 neurons with ReLU activation, which decreases the feature number while maintaining the main patterns. A thick final layer with four neurons and SoftMax works by splitting each picture into one of the defined maize leaf disease categories.

By combining CNN's and ViTs, the hybrid model demonstrates superior classification accuracy compared to standalone architectures, effectively balancing computational efficiency and diagnostic precision. The integration of convolutional and transformer‐based components allows the model to excel in complex image classification tasks by capturing both localized patterns and broader contextual relationships. Figure 12 visually illustrates the hybrid CNN‐ViT architecture, showing how the image passes through convolutional and transformer layers before merging into a final feature representation. This design ensures the model effectively learns long‐range dependencies while preserving fine details, making it highly effective for maize leaf disease detection.

Table 3 gives the parameter details of the hybrid CNN model. Table 3 shows that the result of adding the parameters from the dense layers, the vision transformer block, and the convolutional layers is 87,054,596. This sum accounts for all of the model's trainable parameters; no non‐trainable parameters are included. Convolutional and transformer‐based layers are combined in the hybrid architecture to take advantage of each other's advantages, producing a highly parameterized model that can be applied to challenging picture classification problems.

**TABLE 3:** Hybrid CNN mode parameters.

Layer	Output shape	Parameters
Input (Sequential)	(32, 256, 256, 3, 0)	0
Conv2D – 1	(32, 254, 254, 32)	896
Max‐Pool2D – 1	(32, 127, 127, 32)	0
Conv2D – 2	(32, 125, 125, 64)	18,496
Max‐Pool2D – 2	(32, 62, 62, 64)	0
Conv2D – 3	(32, 60, 60, 64)	36,928
Max‐Pool2D – 3	(32, 30, 30, 64)	0
Conv2D – 4	(32, 28, 28, 64)	36,928
Max‐Pool2D – 4	(32, 14, 14, 64)	0
Conv2D – 5	(32, 12, 12, 64)	36,928
Max‐Pool2D‐ 5	(32, 6, 6, 64)	0
Conv2D – 6	(32, 4, 4, 64)	36,928
Max‐Pool2D‐ 6	(32, 2, 2, 64)	0
Dropout (0.5)	(32, 2, 2, 64)	0
Flatten	(32, 256)	0
ViT Block		
Vision Transformer (ViT)	(32, 768)	86,870,784
Concatenation	(32, 1024)	0
Dense	(32, 64)	177,280
Dense (Output)	(32, 4)	260

*Note:* CNN model parameters: 183,812. ViT model parameters: 86,870,784. Total Parameters = 87,054,596. Trainable Parameters = 87,054,596. Non‐Trainable Parameters = 0.

By using the Hybrid CNN‐ViT model, maize disease detection becomes faster and more accurate, resulting better and more predictable crop results and increased food security. Enabling quick action to control outbreaks, it lowers the risk of huge maize losses and maintains food stability, mainly in areas that make use of large numbers of maize. The model limits overuse of pesticides, as it helps control diseases specifically, saves the environment and supports healthy soil. Combining mobile apps, IoT sensors and drones into agriculture supports efficient monitoring of widespread diseases and makes farming more based on data. The model limits overuse of pesticides, as it helps control diseases specifically, saves the environment, and supports healthy soil. Connected technology from mobile apps, IoT devices and drones improves disease surveillance in the agriculture industry.

## 4 Results and Discussion

This section presents a detailed evaluation of the base CNN and proposed hybrid CNN‐ViT model for maize leaf disease classification. The analysis includes performance comparison with baseline models, ablation studies on architectural configurations, and an assessment of the optimization strategy and hyperparameter selection.

### 4.1 Optimization Strategy and Hyperparameter Impact Analysis

This study was implemented using TensorFlow and Keras, a widely adopted deep learning framework known for its flexibility and scalability in designing and training complex neural network architectures. The training process was carried out in a GPU‐accelerated environment to leverage high‐performance computing for efficient processing particularly due to the increased complexity introduced by the integration of the ViT with the CNN architecture.

To optimize training, a batch size of 64 was selected based on empirical tuning, and a dropout rate of 0.5 was employed to mitigate overfitting by randomly deactivating neurons during training. The model was trained using the RAdam optimizer, an enhanced variant of the Adam optimizer that introduces a rectification mechanism to address the variance of adaptive learning rates during the early stages of training. This rectification allows for more stable convergence, especially in architectures that combine convolutional and transformer‐based components. An initial learning rate of 0.0001 was chosen based on both literature support and empirical evaluation. A manual grid search was performed to assess the impact of key hyperparameters, including learning rates (1e‐4, 5e‐4, 1e‐3), batch sizes (16, 32, 64), and dropout rates (0.3, 0.5). Among the tested optimizers (Adam, RMSProp, and RAdam), RAdam consistently delivered faster convergence and better final validation accuracy. Although a comprehensive sensitivity analysis is beyond the scope of this study, the model demonstrated stable performance under small perturbations in learning rate (± 1e‐4), indicating robustness to minor parameter variations. The training objective was achieved using the sparse categorical cross‐entropy loss function, suitable for multi‐class classification with integer‐encoded labels. A summary of the selected training parameters and hyperparameters is provided in Table 4.

**TABLE 4:** Hyperparameter tuning.

S.No	Simulation hyperparameter	Value
1.	Learning rate	0.0001
2.	Batch size	64
3.	Epoch	30
4.	Optimizer	RAdam
5.	Loss function	Sparse categorical cross entropy
6.	Dropout	0.5

### 4.2 Result Analysis for the Base CNN Model

The CNN model is evaluated based on the accuracy and loss curves recorded at the time of model training and validation. A further performance matrix is also drawn to examine the model's accuracy. Table 5 presents the details of the accuracy and loss development during training and validation. From Table 6, the training and validation metrics can be observed during a 30‐epoch period. The model began the first epoch with a high training accuracy (T.A) of 67.93% and a training loss (T.L) of 0.75, while the validation accuracy (V.A) was 80.18%, and the validation loss (V.L) was 0.80. This means that although it generalizes better on previously unseen data, it has still learned large mistakes on the training set very early. The model improved substantially, with its training and validation accuracy at epoch 5 being 87.69% and 87.80%, respectively, with lower training and validation losses than those in the previous plot. While training continued, at epoch 10, the T.A increased to 92.15%, but the V.A slightly decreased to 86.89%, while the V.L became slightly higher, equaling 0.43, which may indicate the presence of some degree of overfitting. However, by epoch 15, the model generally became stronger in terms of generalizability, reflected by a notable decrease in the V.L to 0.16 and an increase in its V.A to 93.99%. It reaches 95.43% by epoch 20, while the T.A reaches 98.56%, indicating good performance with minor variations in losses. While approaching the end, by epoch 25, the model is close to a perfect T.A of 99.10%, with the accuracy of its validation continuing to improve and reaching 96.97%. During the last epoch, the V.A surpasses 98%, while the T.A stabilizes at 99.16%, and the V.L is as low as 0.11. It learns well, but it does not overfit; it shows steady growth in both the training and validation sets. At the end of the training procedure, high accuracy with low error rates is achieved.

**TABLE 5:** Loss and accuracy development for base CNN model.

Epoch	Training loss (T.L)	Training accuracy (T.A)	Validation loss (V.L)	Validation accuracy (V.A)
1	0.75	67.93	0.80	80.18
5	0.32	87.69	0.31	87.80
10	0.19	92.15	0.43	86.89
15	0.10	95.70	0.16	93.99
20	0.04	98.56	0.19	95.43
25	0.03	99.10	0.15	96.97
30	0.02	99.16	0.11	98.01

**TABLE 6:** Performance parameters for the base‐CNN model.

Class	Precision %	Recall %	F‐1 score %	Accuracy %
0	98.09	98.12	98.11	98.01
1	97.53	98.50	98.31	
2	98.98	97.51	98.24	
3	98.11	98.50	98.26	

**FIGURE 14:**
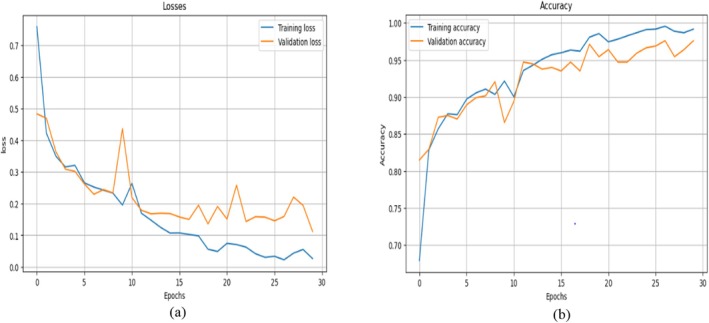
Results of the base CNN model: (a) Training and validation loss and (b) training and validation accuracy.

Figure 14a,b shows a graphical representation of the model loss and accuracy. Figure 14a shows that the model T.L gradually decreases with slight fluctuations in its value, whereas the V.L decreases at the start of training but increases between the 7th and 10th epochs. Then, it decreases gradually; as the training reaches its end epoch, the V.L increases, but at the end epoch, it decreases to a consistent value.

Figure 14b displays the base CNN model accuracy development during training and validation. The figure shows that both accuracy values increase swiftly as the training continues. Slight variations in V.A can be seen, but then it increases and reaches a very good value of 98.01%. The graph shows that the training of the model was smooth and that the model learned the features of the data efficiently. This section displays the performance parameters and confusion matrix achieved during the testing of the model. Table 6 summarizes the results of the accuracy, precision, recall, F1 score, and recall for each class using the CNN model. This provides insights into how well the model performs in its predictions over any one class with an assurance not to be biased toward false positives and false negatives.

**FIGURE 15:**
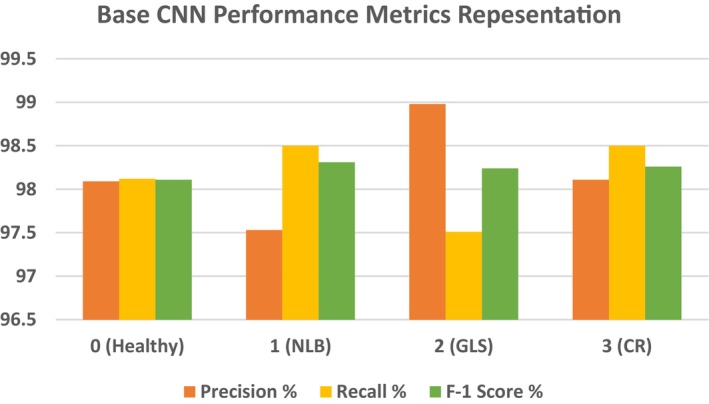
Development of performance metrics for the base CNN model.

Table 6 shows that the precision for Class 0 is 98.09%, which means that 98.09% of those cases in which the model predicted as Class 0 were originally Class 0. This high percentage indicates that very few false positive predictions for this class are made by the model, although the few misclassified cases might have been mistakenly labeled as Class 0. The model manages to identify with high recall, 98.12%, how many actual instances of Class 0 were present. Owing to its high recall, this model almost always captures most real Class 0 examples by effectively reducing false negatives. The F1 score of 98.11% provides a balance between recall and precision. The F1 score of Class 0 is very good, reflecting excellent overall performance in avoiding false negatives while correctly predicting true positives. The precision of Class 1 is 97.53%, which means that 97.53% of the predictions given for Class 1 are correct. Although the model correctly predicts Class 1, there seems to be a slightly larger rate of false positives than other classes do, as depicted by a modest decline in recall. The model managed to correct 98.50% of all real Class 1 examples, leaving only a very few out. The F1 score of 98.31% demonstrates the strong performance of the model in recognizing and classifying images of Class 1, with a slight bias toward higher recall. The model predicts Class 2 with an incredibly high precision of 98.98% for Class 3. This means that very few false positives were present. Recall: 97.51% Class 2 recall is lower than precision compared with other classes, which means that the model missed fewer true Class 2 occurrences and produced a slightly greater quantity of false negatives. F1 stands at 98.24%. When the recall slightly decreases, the F1 score remains high, which means that the performance is good in finding a good balance between the recall and precision. For Class 3, the precision value is 98.11%, the recall is 98.50%, and the f‐1 score is 98.2. These values demonstrate the high model's ability to classify maize diseases. Figure 15 shows a graphical view of the precision, recall and F‐1 score for the CNN model.

**FIGURE 16:**
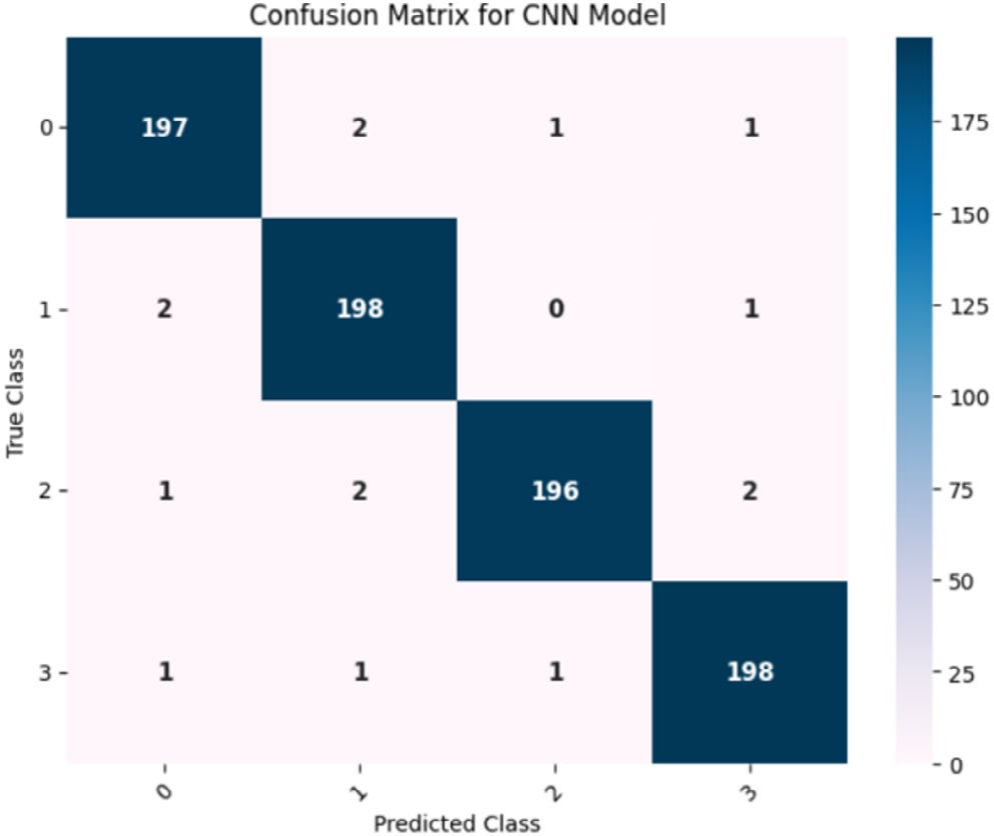
Confusion matrix for the base CNN model.

Figure 16 confusion matrix illustrates how well a CNN model performed in classifying the different maize leaf diseases into four separate groups. Here, the intended class is shown in each column, whereas the actual class is shown in each row. The correct predictions for each class are represented by the entries (197, 198, 196, and 198) on the main diagonal, which constitute the entries from the upper left to the lower right. This finding indicates that the model successfully predicted the majority of the samples. Among the samples classified as Class 0, 197 were correctly classified as such, whereas 2 were incorrectly classified as Classes 1 and 1 as each of Classes 2 and 3. Furthermore, of the 198 samples that were accurately identified, only two from class 1 were incorrectly classified as class 0, and one from class 3. In contrast, Class 2 shows that 196 samples were correctly identified, 2 samples for Class 1 were incorrectly classified, and 1 sample each for Classes 0 and 3. In contrast, just one sample was incorrectly identified as Class 0, one as Class 1, and one as Class 2. Class 3 contained 198 samples that were correctly classified. The model appears to have good recall and precision overall, as indicated by the low percentage of misclassifications.

**FIGURE 17:**
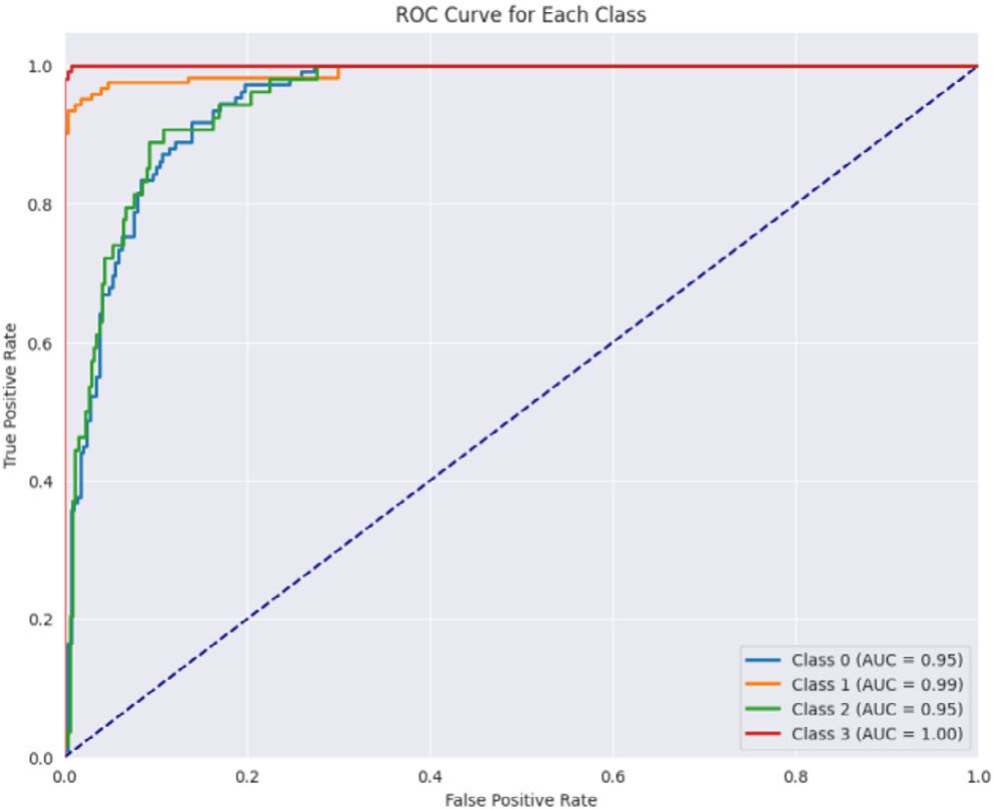
ROC curve for the base CNN model.

Figure 16 displays the confusion matrix achieved during the testing phase of the model. This figure shows that out of 804 test images, 789 images are correctly predicted. Figure 17 showcases the ROC curve achieved for the Base CNN model. The ROC curve for each class illustrates how well the model differentiates between positive and negative samples at various thresholds. Class 0 shows a strong performance with an AUC of 0.95, indicating that the model is good at identifying this class. Class 1 performs even better with an AUC of 0.99, reflecting excellent classification ability. Class 2 has a slightly lower AUC of 0.95, still showing good performance but with a more gradual increase in the true positive rate. Class 3 stands out with a perfect AUC of 1.00, meaning the model perfectly classifies this class without any errors. The dashed diagonal line represents random chance, and since all the class curves are above it, it confirms that the model is performing better than random, with some classes reaching near‐perfect performance.

### 4.3 Result Analysis for the Hybrid CNN‐ViT Model

An overview of the process of training the model is given in Table 7, which represents the entire process of 30 epochs of training, indicating several important performance indices: T.A, T.L, V.L, and V.A. Initially, the model is still learning and fine‐tuning its parameters, as indicated by the early T.A of 78.84% at epoch 1 and a T.L of 0.52. This is further supported by the very high value of the V.L of 1.31 and the poor V.A of 54.29%, which indicate that, at the very beginning, the model generalizes poorly to new data. At epoch 5, the model starts to learn efficiently and starts to generalize better. This is substantiated by a large decline in the training and validation losses and an improvement in T.A of 90.99% and V.A of 88.92%. This means that the training and validation losses regularly decrease as the training enters the middle stage between epochs 10 and 20. At the 20th epoch, the accuracy of training reaches 98.43%, with the loss of training dropping to 0.05, meaning that the model makes relatively few mistakes on this training set. Moreover, the V.A increases to 98.09%, and the V.L decreases to 0.09. These findings reflect the good generalization ability of the model. However, at epoch 25, the T.A increases to 98.67%, with the T.L decreasing to 0.03 at that point. At epoch 30, however, it is slightly different in that the V.L increases to 0.16, whereas the V.A is as high as 99.15%. However, the T.A continues to improve and reaches 99.22%. This could mean that a minor overfitting model becomes increasingly reliant on the training set with variable generalization to new data. In summary, this model is very consistent across the course of epochs: it constantly reduces both training and validation losses, while at the same time largely increasing accuracy on both datasets. This shows excellent generalization performance given that the V.A was high at epoch 30, but this small rise in the V.L would probably mean that overfitting may just be kicking in. Early stopping or regularization might therefore be considered to avoid this. On the whole, by the end, it performs an extremely good job, which makes it highly effective to deploy, with careful monitoring for overfitting.

**TABLE 7:** Loss and accuracy development for hybrid CNN‐ViT model.

Epoch	Training loss (T.L)	Training accuracy (T.A)	Validation loss (V.L)	Validation accuracy (V.A)
1	0.52	78.84	1.31	54.29
5	0.23	90.99	0.38	88.92
10	0.12	95.43	0.25	93.60
15	0.08	97.17	0.11	96.93
20	0.05	98.43	0.09	98.09
25	0.03	98.67	0.03	98.77
30	0.02	99.22	0.16	99.15

**FIGURE 18:**
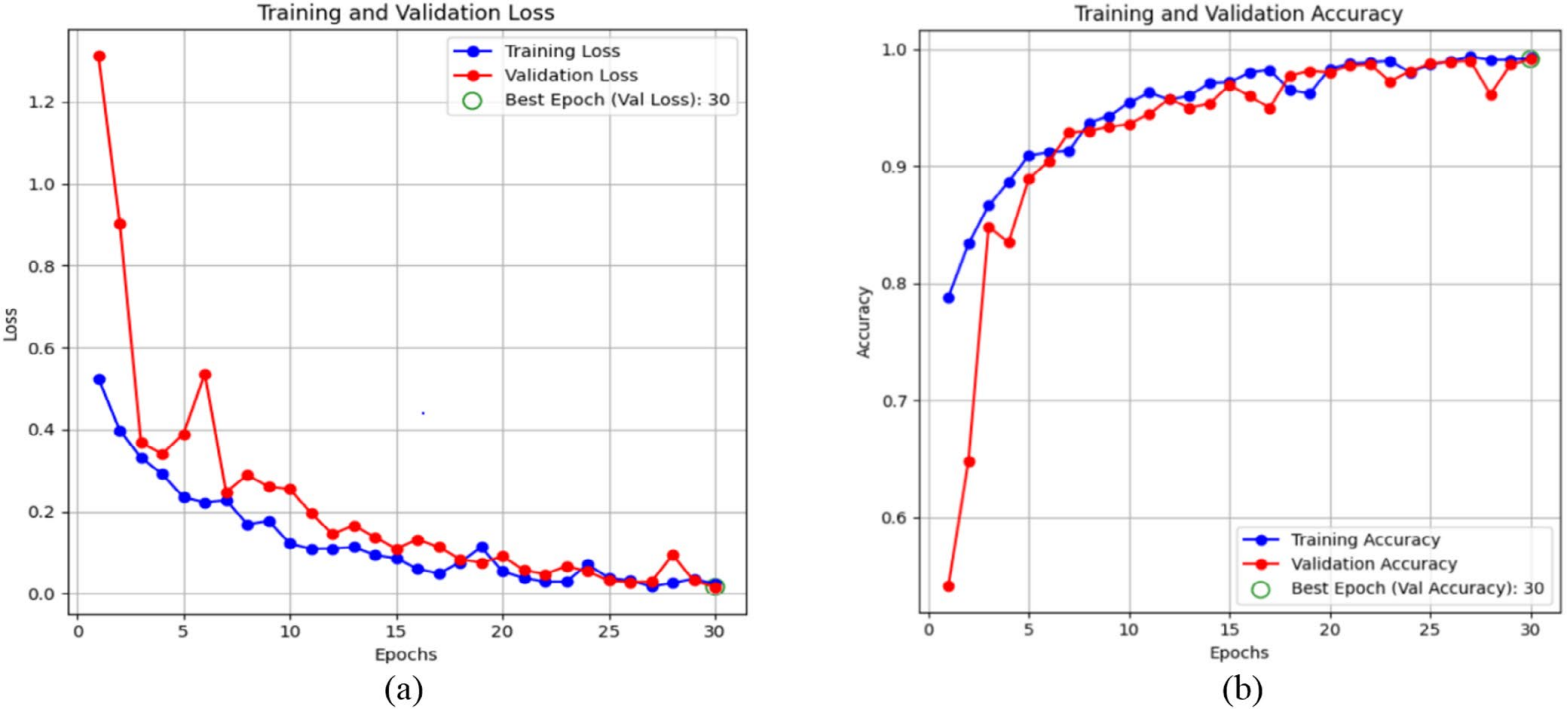
Graphical representation of hybrid CNN‐ViT model (a) loss and (b) accuracy.

Figure 18a,b Shows the hybrid‐CNN model's accuracy and loss of graphical representation. Figure 18a displays the graph of training and validation loss, which demonstrates that the loss decreased within the model. While the model minimizes the errors in the training data, the T.L tends to decrease as time progresses. A similar downward trend is replicated in the V.L, although a few early oscillations are common when the model changes its weights. It is at that moment when the model best generalizes, given that the green circle highlights a point with the lowest V.L during the 30th epoch when both training and validation losses are very low. Figure 18b shows the graph of training and validation accuracy; this accuracy tends to rise with a gradual slope across the epochs. At the beginning, the T.A is low, but as the model gradually gains some experience, it increases greatly to nearly 100% around the 30th epoch. For the V.A, the pattern is somewhat similar; after starting slightly lower than the training set, it gradually improves before leveling at approximately 99%. The epoch represented by the green circle is that at which the V.A has been highest and is thus likely to be the optimum at which the model should operate on unobserved data. This does not appear to be a case of significant overfitting; the model generalizes well and does not just memorize the training set, as the training and validation accuracies remain closely coupled through the epochs.

Table 8 displays the performance matrix recorded during the testing phase of the Hybrid‐CNN model. The table below depicts the performance metrics of the four classes of the classification model. Class 0 had a high precision of 99.51%, which indicates that the forecasts are mostly accurate in that class. With a recall of 99.02%, the model demonstrated the ability to find most of the cases in this class to be correct. The F1 score of Class 0 was 99.25%, which represents the balance between recall and precision. Class 1 had a slightly lower precision, 98.52%, which means that the positive predictions that the model made for this class were not that correct. On the other hand, Class 1 had an excellent recall rate of 99.50%, which for most matters and purposes, practically all incidents were recorded by the model. This result combined for a very strong F1 score of 99.01%. The recall of Class 2 was 98.50%; hence, only a few of the actual cases were missed by the model. On the other hand, the precision of the model was 99.49%, which suggested a high number of valid positive predictions. However, the overall performance was very strong, as noted by an F1 score of 99.0%. In the end, Class 3 had a balanced performance, with a 99.0% recall and a precision of 99.51%. Class 3 had a 99.25% F1 score, which means that the model had approximately equal ability in the identification and prediction of that class. Indeed, it was this fact that it always showed very high accuracy and reliability in such tasks of classification for all classes, with the F1 scores for each class all circling 99%, which furthered the resilience of the model in such a way. The overall accuracy achieved is 99.15% as shown in Table 8.

**TABLE 8:** Performance metrics for hybrid CNN‐ViT model.

Class	Precision %	Recall %	F‐1 score %	Accuracy %
0	99.51	99.02	99.25	99.15
1	98.52	99.50	99.01	
2	99.49	98.50	99.0	
3	99.0	99.51	99.25	

**FIGURE 19:**
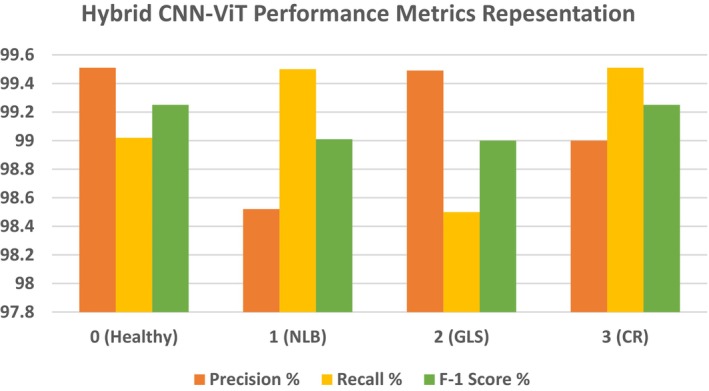
Graphical presentation of performance metrics for the Hybrid‐CNN‐ViT model.

**FIGURE 20:**
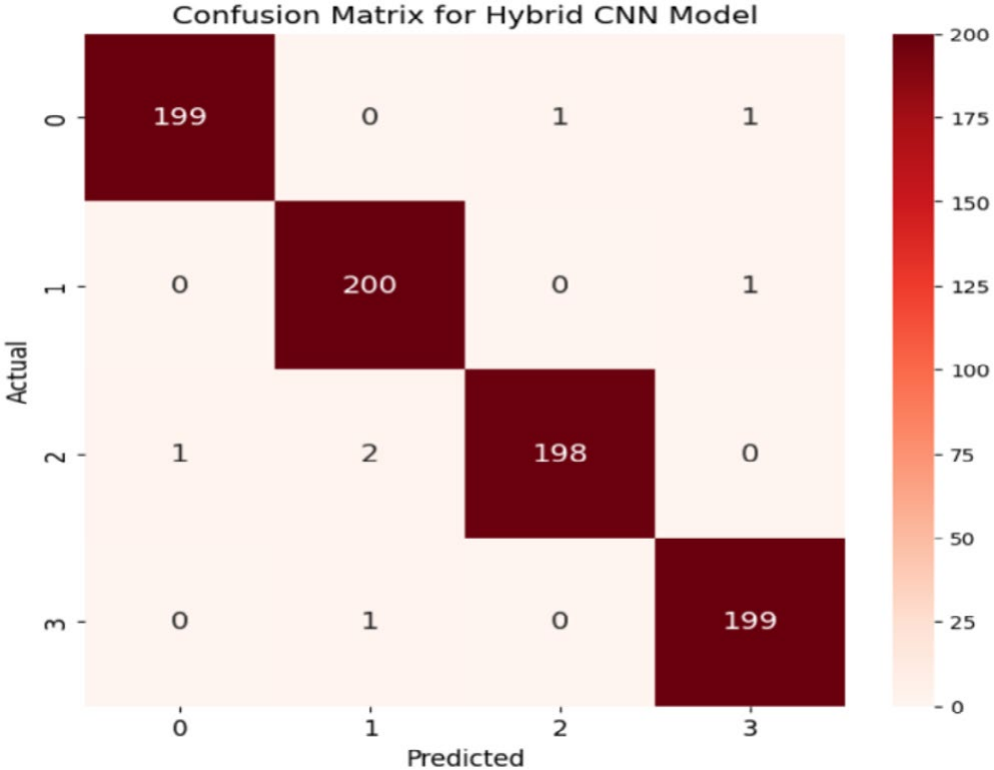
Confusion matrix for the hybrid CNN‐ViT model.

Figure 19 and Table 8 show precision, recall, and F1‐scores for all classes for the Hybrid CNN Model. This highlights that the model performs highly in many measures. With both Class 0 and Class 3, the precision is extremely high at a value of approximately 99.4%; this points toward a reliable positive prediction by the model of these two classes. Class 1 and Class 2 have less precision, amounting to 98.6% since there are more false positive examples in the classification. Moreover, Class 0 and Class 2 have relatively lower recalls, which means that some of these examples are overlooked in those positive instances. In contrast, represented in green, the recall is for Class 1 and Class 3, meaning that in these classes, the model detects most of the real positive instances. The F1 score, which is the balance between recall and precision for all classes, remains almost invariable, averaging approximately 99%. On the other hand, Class 3 shows the opposite pattern, with high recall but lower precision, whereas Class 0 depicts a trade‐off between high precision and lower recall. This balance is again flipped between Class 1 and Class 2, with Class 1 tending to favor recall and Class 2 tending to favor precision. While the model works well overall, there are some differences between the classes in how they handle false positives versus missed positive predictions. In some instances, it might be possible to optimize both precision and recall. Figure 20 shows the confusion matrix, which indicates that out of 804 test images, 797 are correctly predicted.

**FIGURE 21:**
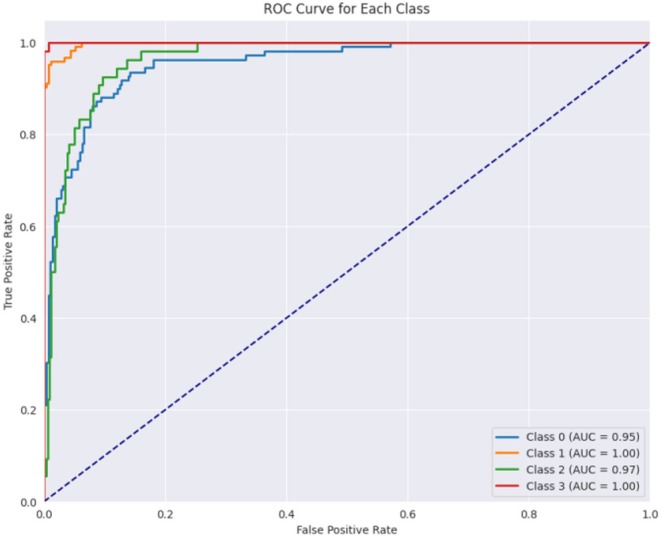
ROC curve for the proposed hybrid CNN model.

Figure 21 displays the ROC curve achieved for the proposed hybrid CNN‐ViT model. From Figure 21 it can be seen that class 0 performs well with an AUC of 0.95, indicating effective identification of this class. Class 1 achieves a perfect AUC of 1.00, demonstrating flawless classification. Class 2 also performs strongly with an AUC of 0.97, slightly below class 1. Class 3 reaches a perfect AUC of 1.00, indicating perfect classification. The dashed blue line represents random performance, and the curves above it confirm that the model performs significantly better than random, with classes 1 and 3 showing near‐perfect results.

### 4.4 Performance Comparison of the Base CNN and Hybrid CNN‐ViT Models

This section evaluates and compares the recall and accuracy, precision, and f‐1 score results between both models. A comparative analysis of model efficiencies based on performance parameters appears in Table 9. According to the provided table, the hybrid CNN‐ViT model achieved superior results compared to CNN across all performance measurement points. The model's accuracy of 99.15%, overall precision of 99.13%, recall of 99.13%, and f‐1 score of 99.12% are exceptionally high.

**TABLE 9:** Comparative analysis of model performance for base‐CNN and hybrid CNN‐ViT model.

Models	CNN	Hybrid CNN‐ViT
Accuracy (%)	98.01	99.15
Precision (%)	98.17	99.13
Recall (%)	98.15	99.13
F‐1 score (%)	98.23	99.12

**FIGURE 22:**
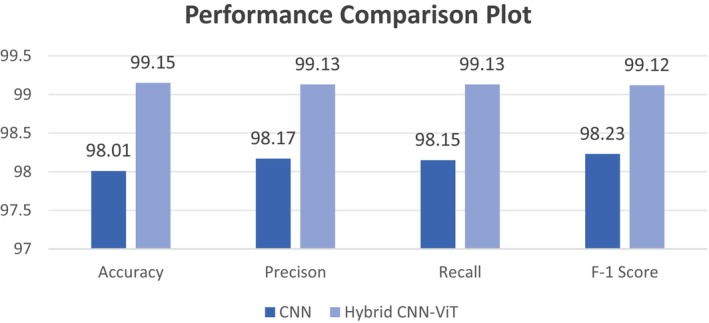
Comparative analysis graphical representation.

Figure 22 displays the graphical presentation of the comparison parameters on which the model's performance was compared. The hybrid model outperforms the CNN with an accuracy of 99.15%, which is higher than 98.01% and shows that it can properly categorize most of the data in the correct manner. Precision, which gauges the proportion of accurately anticipated positive cases, displays a comparable pattern, with the hybrid model scoring 99.13% as opposed to 98.17% for the CNN, indicating fewer false positives. Recall measures how well the model can identify true positives, and the hybrid model performs better than the CNN with a score of 99.13% compared with 98.15%. Finally, the hybrid CNN‐ViT has a higher F‐1 score (99.12%) than does the CNN (98.23%), which compares the two systems in terms of accuracy and recall.

This comparison shows that the hybrid CNN‐ViT model outperforms the traditional CNN in terms of robustness and effectiveness. By adding components of Vision Transformer, the hybrid model is probably better able to identify overall trends in the data, which leads to classification results that are more balanced, accurate, and exact. In complicated picture classification tasks, this emphasizes the advantages of integrating ViT's global context processing with CNN's local feature extraction. Table 10 presents the differences between the CNN and hybrid CNN‐ViT models. Table 9 lists the advantages of employing the hybrid CNN‐ViT model over the base CNN model.

**TABLE 10:** Comparative difference between CNN and the proposed hybrid CNN‐ViT model.

Aspect	Base CNN	Hybrid CNN‐ViT
Architecture	Convolutional layers, pooling layers, fully connected layers	CNN backbone for feature extraction, followed by Vision Transformer
Context understanding	Primarily local context with limited global information	Captures both local and global context through self‐attention
Receptive field	Fixed by the size of convolutional filters and pooling	Flexible and can dynamically focus on different parts of the image
Computational complexity	It is lower, depending on the depth and filter size	Higher due to self‐attention mechanisms in Vision Transformer
Training time	Generally, shorter due to lower complexity	Longer due to the complexity of self‐attention in Vision Transformer
Performance	Strong in capturing local features; can struggle with global context	This model achieves superior performance by leveraging both local and global features
Model size	Typically, smaller due to fewer parameters	It is larger due to the additional parameters in the Vision Transformer

### 4.5 Visualization of Classification Results

**FIGURE 23:**
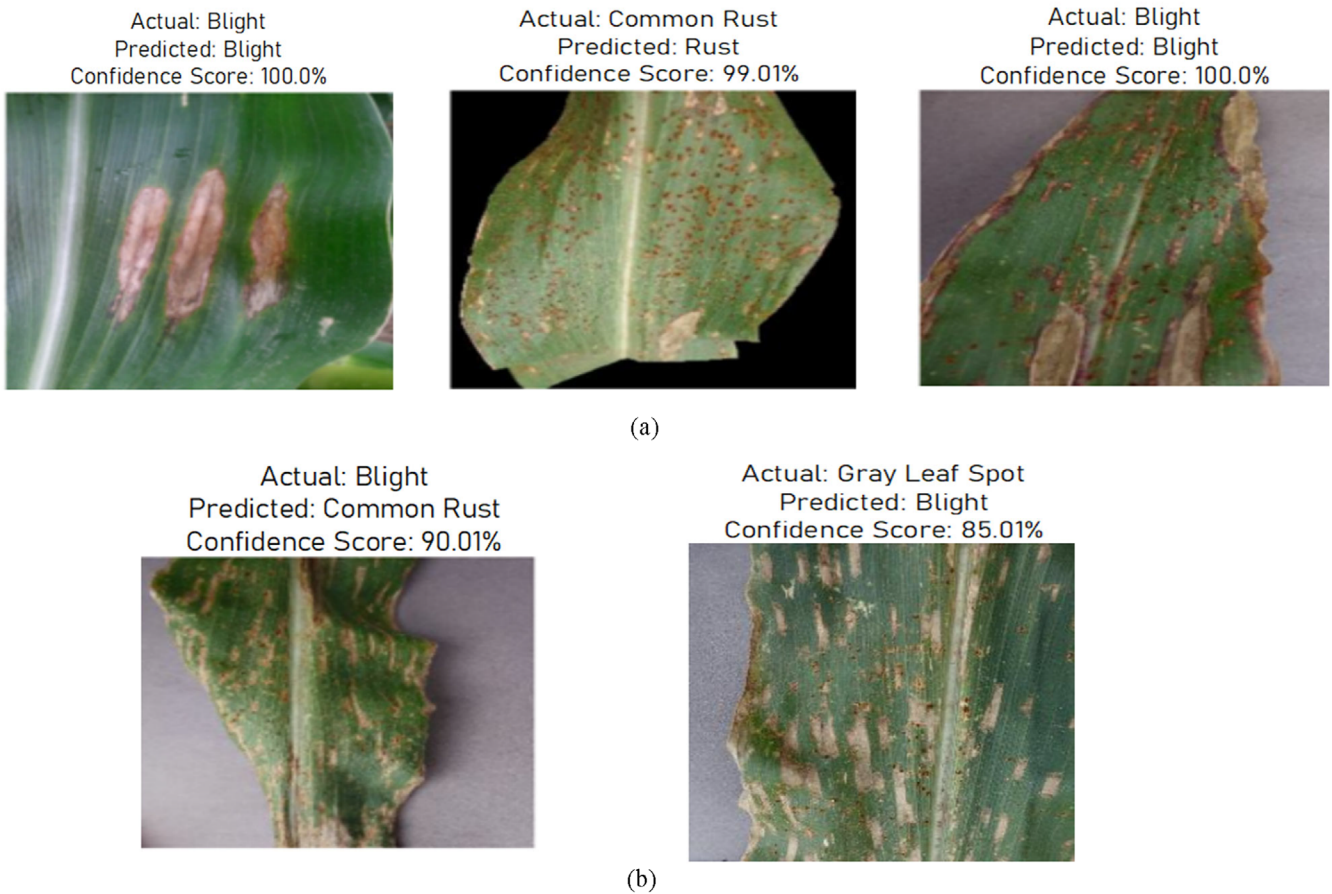
Visualization of classification results (a) correct classified results and (b) miss‐classified results.

This section displays the classification results achieved during the testing of the proposed model. Figure 23 shows the correct classification and miss‐classification results. The misclassification in Figure 23a,b might be due to the model's inability to distinguish subtle differences between Blight and Gray Leaf Spot, especially if these diseases share similar patterns. To improve model performance, increasing the dataset's diversity by adding more annotated examples of both diseases would help the model to differentiate between them more effectively. Additionally, the model could be confused by varying conditions like lighting, leaf texture, or resolution in the images. To address this, enhancing data preprocessing with techniques such as normalization and image enhancement could help it generalize better and reduce such misclassifications.

**FIGURE 24:**
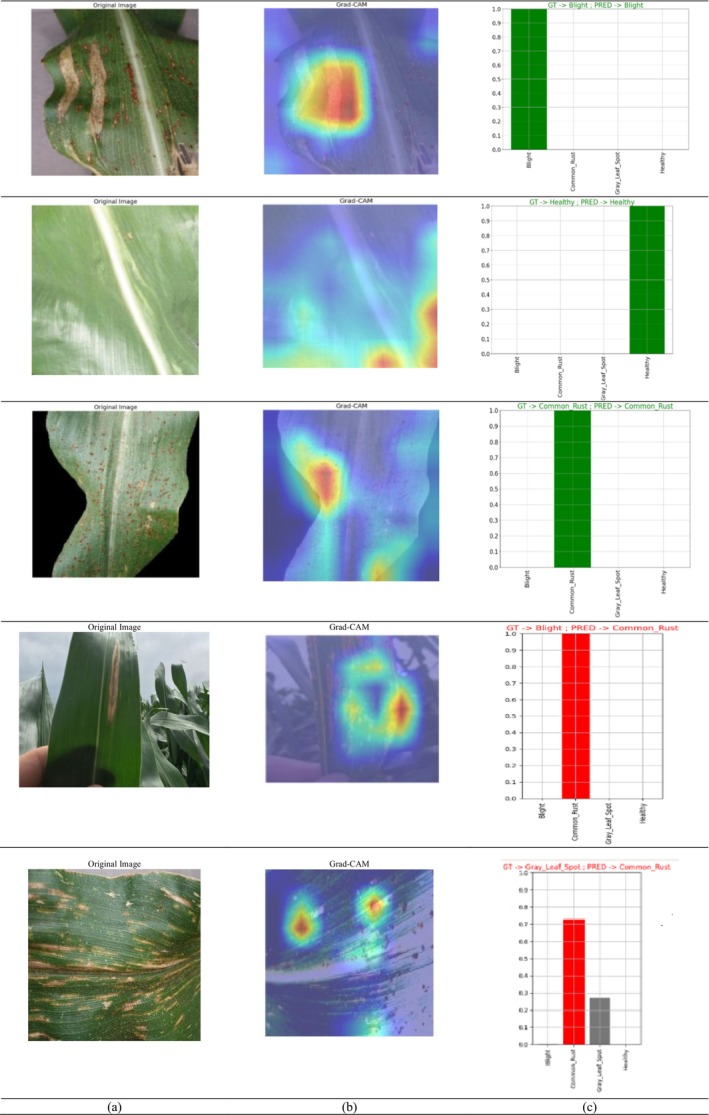
Grad‐CAM visualization results (a) original image, (b) Grad‐CAM, and (c) GT‐predicted graph.

Figure 24 displays the Grad‐CAM visualization. In the top row, Figure 24a displays the original image and Figure 24b displays the Grad‐CAM visualization of the image. Figure 24c displays the graph which compares the actual (ground truth) labels with the predicted labels. The vertical axis (GT) represents the true classes, while the horizontal axis (PRED) represents the predicted classes. From these images, it can be seen that the model is classifying the disease images and healthy maize leaf images effectively.

During the Grad‐CAM‐based visual analysis, it was noted that the proposed Hybrid CNN‐ViT model exhibited minor misclassifications between the gray leaf spot (GLS) and blight classes. This reflects the inherent visual similarity between the two disease types. Both GLS and Blight often present with overlapping symptomatic features such as necrotic lesions, brownish discoloration, and irregular margins, which can be difficult to distinguish even by human experts, especially in the early or transitional stages of infection. The slight confusion observed in some cases highlights the natural challenge of distinguishing between biologically similar patterns rather than a limitation of the model architecture itself. Importantly, the model consistently achieved high precision and recall across all classes, including GLS and Blight, demonstrating its robust overall performance. The inclusion of both laboratory‐curated (Kaggle) and field‐condition (Mendeley) datasets further increased the model's exposure to real‐world variability such as lighting conditions, leaf occlusions, and background complexity. While this enhances generalization, it can also introduce subtle ambiguities that contribute to occasional class overlap.

### 4.6 Class‐Wise Confidence Calibration and ECE Evaluation

**FIGURE 25:**
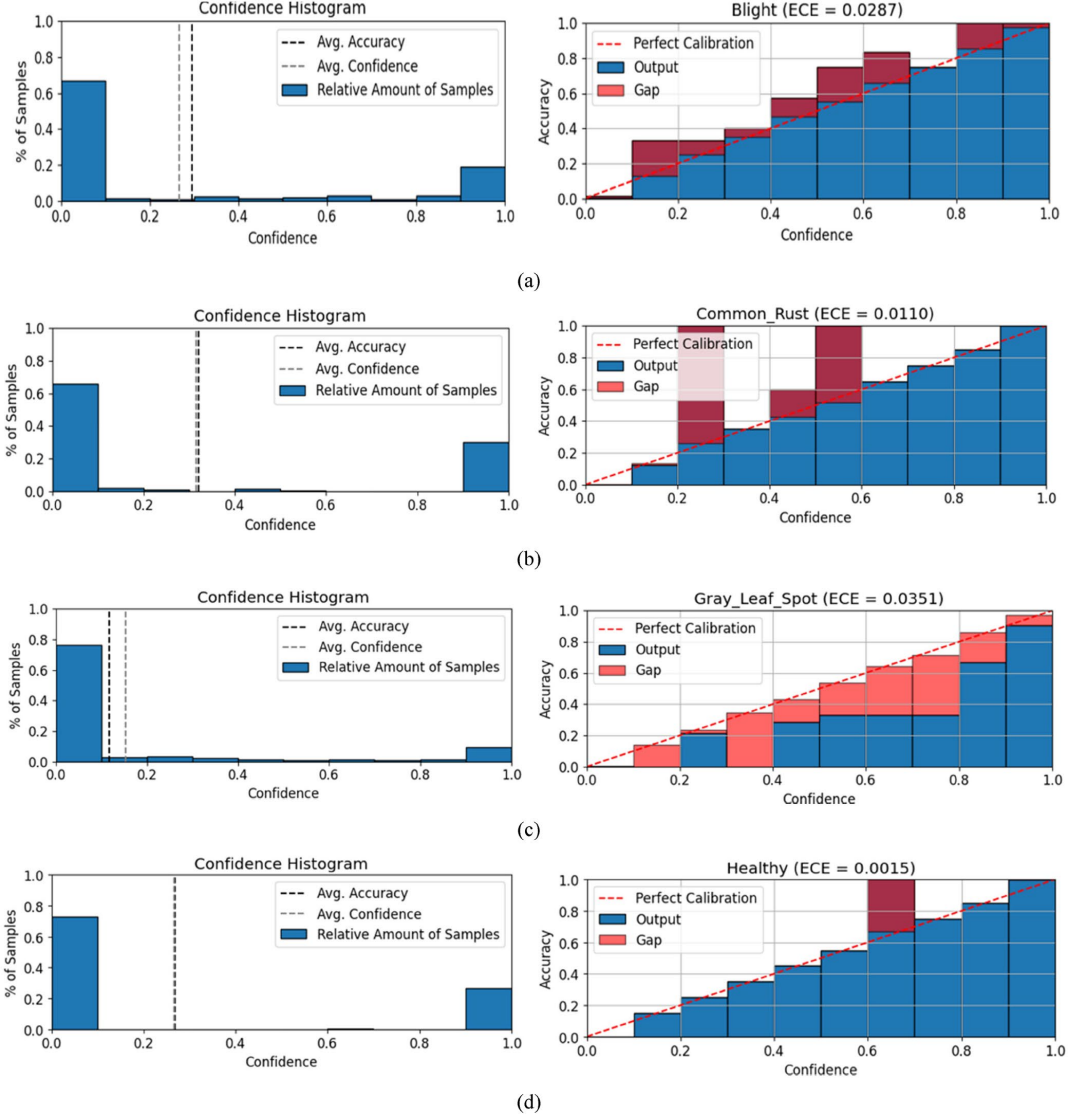
Class‐wise confidence calibration and reliability analysis of the hybrid CNN‐ViT model.

The class‐wise reliability diagrams shown in Figure 25 provide a comprehensive evaluation of the confidence calibration performance of the proposed Hybrid CNN‐ViT model across all four disease categories: blight, common rust, gray leaf spot, and healthy. These diagrams consist of two plots per class; the upper histogram shows the distribution of predicted confidence scores, while the lower plot compares the predicted confidence with the actual accuracy through a reliability curve. The Expected Calibration Error (ECE) is reported for each class, where lower values indicate better alignment between model confidence and observed accuracy.

For the Blight class, the model displays a low ECE of 0.0287, indicating a reasonable level of calibration. The majority of the predictions for this class are concentrated in the lower confidence bins, and the reliability diagram reveals minor overconfidence in certain mid‐confidence regions. In the case of common rust, the model demonstrates excellent calibration, with an ECE of just 0.0110. The reliability diagram for this class closely follows the ideal calibration line, showing that the model's predictions are both accurate and reliable when it assigns high confidence to this class. The gray leaf spot class, however, shows a slightly higher ECE of 0.0351, which is attributed to greater uncertainty in the model's predictions. This is also reflected in the Grad‐CAM‐based visual analysis, where occasional confusion was noted between gray leaf spot and blight due to their visual similarity. The reliability diagram for this class shows noticeable gaps between predicted confidence and actual accuracy, suggesting slight overconfidence in several intervals.

In contrast, the healthy class achieves near‐perfect calibration with an ECE of 0.0015. The model's predicted confidences for this class align almost exactly with the actual observed accuracies, indicating highly reliable performance. Overall, the reliability diagrams confirm that the proposed hybrid CNN‐ViT model is well‐calibrated across all classes, with particularly strong performance for common rust and healthy categories. The slightly higher ECE for gray leaf spot highlights the need for further refinement in distinguishing visually similar diseases, but does not significantly detract from the model's overall reliability. These findings underscore the model's effectiveness in not only achieving high accuracy but also in providing dependable confidence estimates for real‐world agricultural decision‐making.

### 4.7 Ablation Study: Architecture and Component Evaluation

To assess the impact of architectural components and design choices on the performance of the proposed hybrid CNN‐ViT model for maize leaf disease classification, we conducted a comprehensive ablation study in two stages: (i) comparative evaluation of model types (base CNN, ViT, and hybrid CNN‐ViT), and (ii) structural ablation of different CNN and ViT configurations.

#### 4.7.1 Component‐Level Comparison

To assess the impact of different architectural components on the performance of the hybrid CNN‐ViT model for maize leaf disease classification, an ablation study was conducted. The baseline CNN‐only model was first tested, demonstrating strong local feature extraction but limited global contextual understanding. In the ablation study, we evaluated three models: base CNN, ViT, and hybrid CNN‐ViT. The base CNN achieved 98.01% accuracy with high precision, recall, and F1 scores (~ 98%) and an AUC ≥ 0.95, making it highly effective for smaller datasets. The ViT, with an accuracy of 97.12%, precision of 96.45%, recall of 96.78%, and an F1 score of 96.52%, performed well but showed slightly lower results than the CNN, especially on smaller datasets, although it still maintained an AUC ≥ 0.95. The Hybrid CNN‐ViT model outperformed the others with 99.15% accuracy and nearly perfect precision, recall, and F1 scores (~ 99%). This model benefits from combining CNN's local feature extraction and ViT's global dependency capture, leading to superior performance, with an AUC ≥ 0.98.

Overall, while base CNN works well for smaller datasets and ViT excels with large data, the hybrid approach delivers the best results for complex image classification tasks. From Table 11, it can be seen that the fusion of CNN‐ViT outperforms the Base CNN model in terms of every performance measure.

**TABLE 11:** Component level ablation study.

S.NO	Model used	Accuracy %	Precision %	Recall %	F‐1 Score	ROC
1.	Base CNN	98.01	98.17	98.15	98.12	AUC ≥ 0.95
2.	ViT	97.12	96.45	96.78	96.52	AUC ≥ 0.95
3.	Hybrid CNN‐ViT	99.15	99.13	99.13	99.12	AUC ≥ 0.98

**TABLE 12:** Structural ablation study.

CNN blocks	Transformer depth	Patch size	Attention head	Accuracy %
4	4	16 × 16	6	94.89
6	6	16 × 16	8	99.15
8	8	8 × 8	12	95.23

#### 4.7.2 Structural Ablation: Design Optimization

To optimize the architectural parameters of the Hybrid CNN‐ViT model, we experimented with varying depths for the CNN and transformer components, as well as ViT‐specific parameters such as patch size and attention heads. The results indicated that increasing CNN depth from 4 to 6 blocks significantly improved feature abstraction without overfitting, while moving beyond 6 blocks yielded diminishing returns with increased computational cost. For the transformer component, we selected a patch size of 16 × 16 based on prior studies demonstrating its effectiveness in preserving local–global context. We used 8 attention heads to enhance the model's ability to attend to different spatial representations concurrently. A transformer depth of 6 layers provided the best trade‐off between accuracy and overfitting. Table 12 gives a detailed description for it.

The ablation study, conducted in two phases, validates the design effectiveness of the proposed hybrid CNN‐ViT architecture. The first phase, comparing the base CNN, ViT, and the hybrid model, demonstrated that integrating CNN with ViT leads to superior classification performance by leveraging both local feature extraction and global dependency modeling. The standalone ViT baseline used in the first phase of the ablation followed the same transformer configuration as the hybrid model (patch size = 16 × 16, depth = 6, attention heads = 8) to ensure a fair comparison. The hybrid CNN‐ViT outperformed both individual models across all evaluation metrics, achieving an accuracy of 99.15% and an AUC ≥ 0.98. In the second phase, we systematically evaluated the impact of varying CNN depth and ViT configurations. The six‐block CNN combined with a 6‐layer transformer using 16 × 16 patches and 8 attention heads achieved the best trade‐off between accuracy and calibration performance (ECE), confirming its suitability for the task. These findings highlight the significance of jointly optimizing both CNN and transformer components to build a robust and generalizable model for maize leaf disease classification.

### 4.8 Comparison of the Proposed Hybrid CNN‐ViT Model With Other Transfer Learning Model

A comparison of the proposed hybrid CNN‐ViT model performance occurred against AlexNet, DenseNet121, VGG19, and InceptionV3 transfer learning models when detecting plant diseases in maize has been demonstrated in Table 13. The hybrid CNN‐ViT model achieved better performance than transfer learning models because it extracted local and global features successfully. The hybrid CNN‐ViT model shows an accuracy of 99.15% as it extracts nearby pixel patterns combined with spatial information from its feature extraction capabilities and trained weight metrics. Due to its advantages, this hybrid CNN‐ViT approach beats single‐purpose transfer learning systems at handling difficult tasks.

**TABLE 13:** Comparative evaluation of the proposed model with transfer learning model.

Model name	Accuracy %	Precision %	Recall %	F‐1 score %
DenseNet121	97.35	97.11	96.76	96.35
VGG19	96.99	96.80	96.01	96.40
AlexNet	97.82	96.34	97.02	96.68
InceptionV3	98.65	98.22	98.04	98.13
Proposed hybrid CNN‐ViT	99.15	99.13	99.13	99.12

### 4.9 Comparison Analysis With Current State of Art

To benchmark the effectiveness of the proposed hybrid CNN‐ViT model, a comparative analysis was conducted against several state‐of‐the‐art methods reported in the recent literature for maize (corn) leaf disease classification. These methods include both classical machine learning techniques and modern deep learning architectures such as MobileNet, ResNet18/152, VGG16 with explainability modules, SVM‐KNN hybrids, and ensemble techniques like XGBoost + KNN.

Table 14 summarizes the comparative results across standard evaluation metrics, including accuracy, precision, recall, and F1‐score. As shown, earlier models like MobileNet and SVM‐KNN exhibit relatively lower performance, with accuracy values of 83.37% and 71.44%, respectively. Classical models, despite their simplicity, showed considerable variance, where the XGBoost + KNN combination achieved 98.77% accuracy and an F1‐score of 96.10%. Among deep learning approaches, ResNet18 and ResNet152 demonstrated robust performance, achieving accuracy scores of 98.01% and 98.34%, respectively.

**TABLE 14:** State of art.

Reference/Year	Journal	Technique/Model employed	Number of images/dataset	Number of images	Number of classes	Performance measures %
(Pratama and Pristyanto 2023)/2023	JITK	MobileNet	Corn/Maize leaf disease	4188	4	Accuracy—83.37 Precision—83.22 Recall—82.89 F‐1 score—83.1
(Prakash and Kirubakaran 2023)/2023	Philippine Journal of Science	XGBoost + KNN	Corn/Maize leaf disease	4188	4	Accuracy—98.77 Precision—95.17 Recall—98.03 F‐1 score—96.10
(Solihin et al. 2023)/2023	Elinvo	SVM_KNN	Corn/Maize leaf disease	4188	4	Accuracy—71.44 Precision—70.08 Recall—71.44 F‐1 score—67.1
(Tariq et al. 2024)/2024	Frontiers	VGG16 + augmented with LRP+ Explainable AI	Corn/Maize leaf disease	4188	4	Accuracy—94.67 Precision—92.91
(Wu 2024)/2024	Agronomy	ResNet18	Corn/Maize leaf disease	4188	4	Accuracy—98.01 Precision—95.02 Recall—95.32 F‐1 score—95
(Gopalan et al. 2025)/2025	BMC Plant Biology	Fine‐Tuned ResNet152	Corn/Maize leaf disease	4188	4	Accuracy—98.34 Precision—98.02 Recall—97.45 F‐1 score—97.91
Proposed Hybrid CNN‐ViT Model			Corn/Maize leaf Disease (Kaggle)	4188	4	Accuracy—99.15% Precision—99.13% Recall—99.13% F‐1 score—99.12%
			Maize leaf disease detection	3852		

Recent studies support improved plant disease detection using advanced imaging techniques. In (Zhou et al. 2024), the authors used 3D point clouds for automated phenotyping, aiding leaf trait analysis, and in (Xu et al. 2022) the authors enhanced image clarity with GAN‐based highlight removal, while in (Wang et al. 2025), authors improved image quality under poor conditions using a dehazing network. These methods help strengthen CNN‐ViT models for maize disease classification (Khan et al. 2023).

**FIGURE 26:**
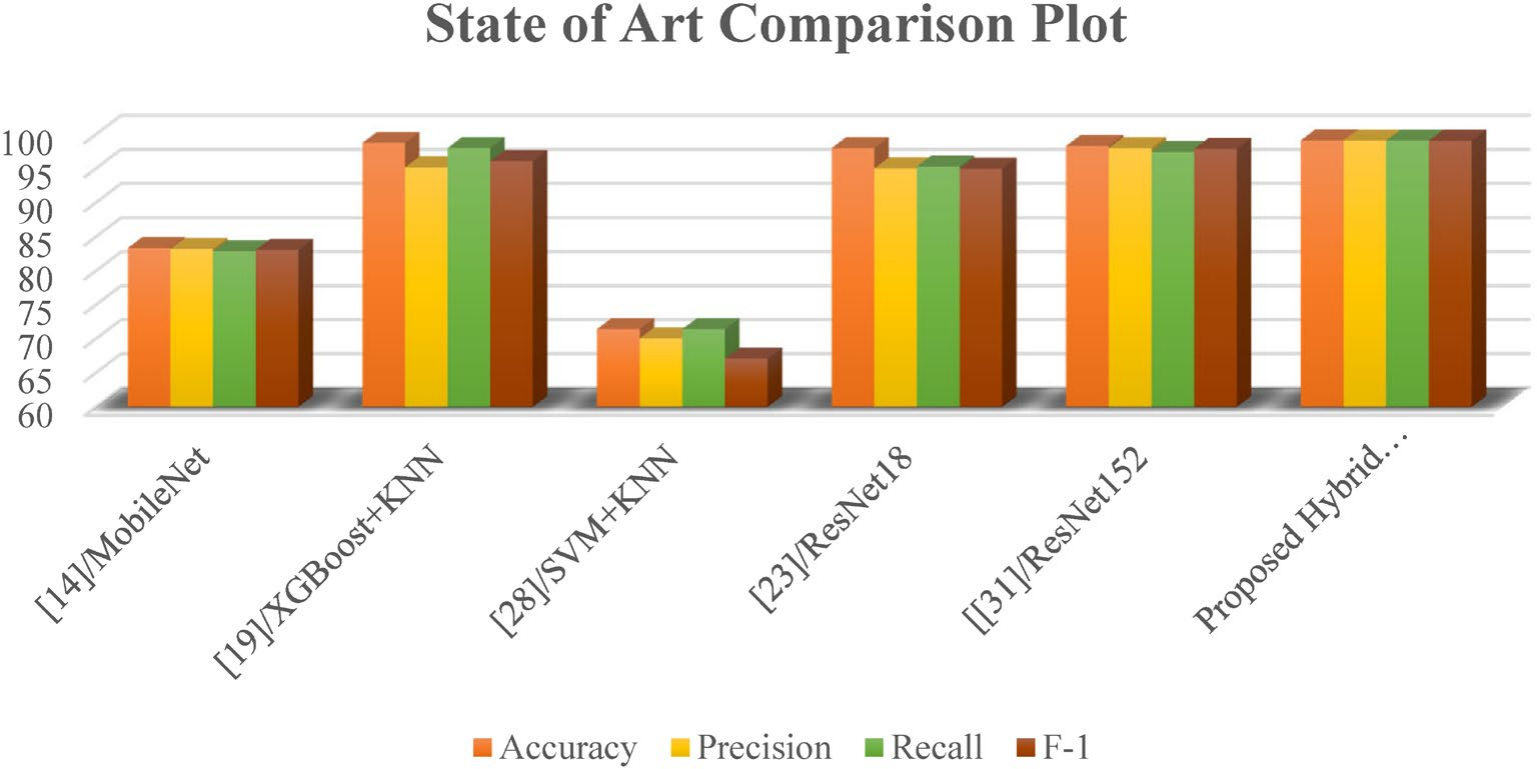
Graphical presentation of comparison analysis.

In contrast, the proposed Hybrid CNN‐ViT model achieved the highest overall performance across all metrics: 99.15% accuracy, 99.13% precision, 99.13% recall, and a 99.12% F1‐score using a combined dataset of 8040 images from publicly available maize leaf disease sources. This performance improvement can be attributed to the architectural synergy between CNNs for local feature extraction and vision transformers for modeling long‐range dependencies. The consistent performance gains across all evaluation criteria highlight the model's robustness and effectiveness for real‐world agricultural disease detection tasks. Figure 26 represents the graphical presentation of the comparative analysis for state of the art models.

### 4.10 Discussion on Real‐Time Deployment Feasibility

The proposed Hybrid CNN‐ViT model demonstrates excellent performance in maize leaf disease classification and has promising potential for integration into IoT‐based agricultural monitoring systems. While it is acknowledged that ViTs introduce additional computational overhead due to the quadratic complexity of their self‐attention mechanism, our current architecture remains within a feasible range for deployment on mid‐tier edge devices, particularly when paired with lightweight convolutional backbones and batch‐wise inference strategies. Furthermore, with minimal architectural adjustments such as reducing transformer depth, optimizing patch size, or replacing the ViT module with its lightweight variants (e.g., MobileViT or Linformer), the model can be efficiently adapted for real‐time inference on resource‐constrained platforms like NVIDIA Jetson Nano, Raspberry Pi 4, or similar edge devices. In future work, we aim to address this challenge by incorporating lightweight transformer designs and applying compression techniques such as quantization and pruning to optimize the model for edge deployment scenarios.

Therefore, while the current model emphasizes accuracy and robustness, it also serves as a scalable foundation that, with modest optimization, can be readily integrated into IoT‐enabled, real‐world agricultural applications.

### 4.11 Computational Profiling and Deployment Feasibility

To assess the practical deployability of the proposed Hybrid CNN‐ViT model, a detailed computational resource profiling was conducted. While the model is designed for high accuracy, it is equally important to evaluate its efficiency in terms of memory, computational cost, and real‐time applicability. The total number of trainable parameters in the hybrid model is 87,054,596, as presented in Table 3. These parameters span the convolutional layers, transformer encoder blocks, and dense layers, making the model highly expressive and capable of learning both local and global visual features. The model's computational complexity was quantified by calculating the floating‐point operations (FLOPs) per inference. For an input image of size 256 × 256 with a batch size of 1, the estimated FLOPs are approximately 10.7 GFLOPs. This makes the model computationally intensive but manageable on modern GPUs and edge inference devices with optimized architectures. During training, the model was executed on an NVIDIA RTX 3090 GPU (24 GB VRAM), where the peak GPU memory consumption was approximately 6.4 GB. During inference, the memory usage was significantly reduced to about 2.1 GB, ensuring that the model can be deployed on mid‐range edge devices like the NVIDIA Jetson AGX Xavier or Jetson Orin with proper optimization.

Furthermore, the inference time per image was recorded at 29.7 milliseconds, enabling a throughput of approximately 34 frames per second (FPS). This speed affirms the model's potential for real‐time deployment in practical applications such as mobile‐based disease detection systems, autonomous agricultural robots, or IoT‐enabled crop monitoring frameworks. This computational profiling confirms that the proposed model balances accuracy with computational feasibility, making it suitable for real‐world agricultural use cases, especially in precision farming and early disease intervention.

### 4.12 Cross‐Dataset Validation and Generalization Study

To assess the robustness and generalization capability of the proposed models, we conducted a 5‐fold cross‐validation on two datasets: (i) the combined Kaggle + Mendeley maize leaf dataset used in this study, and (ii) the corn disease and severity (CD&S) dataset, which offers greater diversity in terms of imaging conditions and disease severity levels. The results are summarized in Table 15. On the original combined dataset, the hybrid CNN‐ViT model consistently outperformed the base CNN, achieving an average accuracy of 99.06%, compared to 97.82% for the base CNN. When evaluated on the CD&S dataset, which was not used in model training, the hybrid CNN‐ViT model again demonstrated strong generalization with an average accuracy of 95.93%, while the base CNN's performance dropped to 89.70%. These results validate the superior generalizability of the hybrid model, especially under varying real‐world imaging conditions. Furthermore, this cross‐dataset setup confirms that the proposed model can maintain high classification performance even when deployed on data from different sources and environments, thereby addressing concerns about dataset‐induced bias and overfitting.

**TABLE 15:** 5‐Fold cross‐validation results for both models on two datasets.

Model	Dataset	Fold	Accuracy %	Precision%	Recall %	F‐1%
Base CNN	Kaggle + Mendeley	Fold 1	97.85	97.80	97.76	97.78
		Fold 2	97.66	97.68	97.62	97.65
		Fold 3	97.92	97.91	97.85	97.88
		Fold 4	97.75	97.70	97.74	97.72
		Fold 5	97.90	97.85	97.82	97.83
Average			97.82	97.79	97.76	97.77
Hybrid CNN‐ViT	Kaggle + Mendeley	Fold 1	99.01	99.00	98.95	98.97
		Fold 2	98.94	98.91	98.90	98.90
		Fold 3	99.15	99.12	99.10	99.11
		Fold 4	99.06	99.04	99.00	99.02
		Fold 5	99.13	99.10	99.08	99.09
Average			99.06	99.03	98.99	99.02
Base CNN	CD&S Dataset	Fold 1	89.25	88.97	88.60	88.78
		Fold 2	89.70	89.50	89.30	89.40
		Fold 3	90.12	89.91	89.80	89.85
		Fold 4	89.85	89.76	89.60	89.68
		Fold 5	89.58	89.30	89.10	89.20
Average			89.70	89.49	89.28	89.38
Hybrid CNN‐ViT	CD&S Dataset	Fold 1	95.84	95.63	95.40	95.51
		Fold 2	96.11	95.90	95.70	95.80
		Fold 3	95.89	95.70	95.50	95.60
		Fold 4	96.04	95.81	95.60	95.70
		Fold 5	95.77	95.56	95.30	95.43
Average			95.93	95.72	95.50	95.60

## 5 Conclusions & Future Scope

This study proposed a hybrid deep learning model that integrates convolutional neural networks (CNNs) and vision transformers (ViTs) for accurate classification of maize leaf diseases. The CNN component effectively captures local spatial features, while the ViT module models long‐range contextual dependencies across the image. The model achieved high performance on the validation set with an accuracy of 99.15%, precision of 99.13%, recall of 99.13%, and F1‐score of 99.13%. To further assess its robustness, we conducted 5‐fold cross‐validation, where the model achieved an average accuracy of 99.06%, precision of 99.03%, recall of 99.01%, and F1‐score of 99.02% on the Kaggle + Mendeley dataset. Moreover, when validated on the independent CD&S dataset, the model maintained strong generalizability, achieving 95.93% accuracy, 95.72% precision, 95.50% recall, and 95.61% F1‐score. These results demonstrate the model's robustness to varying data distributions and its potential for real‐world deployment in field conditions. Future evaluations using real‐time, drone‐based, or live‐streamed imagery will further validate the model's performance under practical deployment scenarios. In conclusion, the proposed CNN‐ViT model advances AI‐driven solutions for plant disease classification by combining high accuracy with scalability, offering a valuable tool for sustainable and intelligent agriculture.

Future research will explore alternative optimization strategies, including knowledge distillation, model pruning, and quantization. Lightweight transformer variants such as MobileViT, TinyViT, and Linformer will be evaluated for their ability to reduce inference time and memory requirements, thereby enabling efficient deployment on edge devices without compromising classification accuracy.

## Author Contributions

**Gunjan Shandilya:** conceptualization (equal), methodology (equal), software (equal), writing – original draft (equal), writing – review and editing (equal). **Sheifali Gupta:** formal analysis (equal), supervision (equal), validation (equal), visualization (equal), writing – review and editing (equal). **Heba G. Mohamed:** formal analysis (equal), funding acquisition (equal), supervision (equal), visualization (equal), writing – original draft (equal). **Salil Bharany:** writing – review and editing (equal), methodology (equal), validation (equal). **Ateeq Ur Rehman:** conceptualization (equal), methodology (equal), writing – review and editing (equal). **Seada Hussen:** data curation (equal), formal analysis (equal), investigation (equal), validation (equal), writing – review and editing (equal).

## Ethics Statement

The authors have nothing to report.

## Consent

The authors have nothing to report.

## Conflicts of Interest

The authors declare no conflicts of interest.

## Data Availability

Datasets used in this study are publically available at https://www.kaggle.com/datasets/smaranjitghose/corn‐or‐maize‐leaf‐disease‐dataset, https://data.mendeley.com/datasets/tywbtsjrjv/1 and https://arxiv.org/abs/2110.12084.
